# Integrated metabolomics and transcriptomics unravel the biosynthZaesis mechanism of anthocyanin in postharvest red raspberry (*Rubus idaeus* L.)

**DOI:** 10.3389/fpls.2025.1549458

**Published:** 2025-05-13

**Authors:** Huajun Sun, Fangzheng Cui, Ying Liu, Lili Qian, Sijing Zhu, Yue Li

**Affiliations:** College of Food Science, Heilongjiang Bayi Agricultural University, Daqing, China

**Keywords:** raspberry, anthocyanin, transcriptome, metabolome, color variation

## Abstract

**Introduction:**

Anthocyanins are crucial secondary metabolites that are responsible for pigment deposition in fruits. Raspberry fruit color shifts from white to red during natural or postharvest ripening. However, the precise mechanisms and biosynthetic pathways of anthocyanins in postharvest raspberries remain unclear.

**Methods:**

This study used metabolomic and transcriptomic analyses to explore anthocyanin biosynthesis in postharvest raspberries at various color stages: white (RBT-1), white-to-pink (RBT-2), pink (RBT-3), red (RBT-4), and deep red (RBT-5).

**Results:**

We identified 43 key metabolites, and 13,239 DEGs linked to anthocyanin biosynthesis in postharvest raspberry colour development, including cyanidin-3-O-sophoroside and cyanidin-3-O-glucoside. The key DAMs in colored raspberries were gentiobioside, pelargonidin-3,5-O-diglucoside, cyanidin-3-O-sambubioside, and pelargonidin-3-O-sambubioside. Transcriptome analysis revealed 32 differentially expressed structural genes linked to anthocyanin and flavonoid synthesis, with significant upregulation of *PAL, CHS, F3H, C4H, F3'H, DFR, ANS, CHI*, and *UFGT* genes, which promote anthocyanin synthesis and pigment accumulation. Integrated analysis showed that cyanidin-3-O-sophoroside was correlated with 9 structural genes involved in anthocyanin biosynthesis, 19 transcription factors (TFs), and 14 hormone signaling-related genes.

**Discussion:**

This study explored the regulatory mechanisms of MYB, WRKY, bHLH, and NAC transcription factors, as well as structural genes and phytohormone-related genes, in modulating anthocyanin metabolism during postharvest color changes in raspberries. The findings provide valuable insights for optimizing postharvest fruit storage conditions and enhancing fruit quality.

## 1 Introduction

The red raspberry (*Rubus idaeus* L.), a perennial deciduous shrub belonging to the genus Rubus of the Rosaceae family, is also known by several other names, including pallet, plucked berry, mulberry, and red berry. It is widely cultivated in South America, North America, Asia, and Europe (Veljković et al., 2019). Red raspberries are a rich source of nutrients, including sugars, fats, proteins, and vitamins. In addition, it is abundant in bioactive components, such as anthocyanins, ellagitannins, salicylic acid, raspberry ketone, and superoxide dismutase. These active substances are believed to resist oxidants (Yu et al., 2015; Kashchenko et al., 2021; Noratto et al., 2017; Zhang et al., 2018), inflammation (Marino et al., 2024b), microbes (Demirbas et al., 2017), and cancer (Chu et al., 2014; Gupta et al., 2019), reducing the occurrence of chronic diseases, including high blood lipid (Jazinaki et al., 2024), blood stress (Jeong et al., 2016), blood sugar (He et al., 2016). Owing to its benefits to health, raspberry was referred to as the “third-generation fruit” and the “golden fruit.” (Ispiryan et al., 2021; Sun et al., 2025).

Anthocyanins are a class of water-soluble pigments widely found in fruits and vegetables and are responsible for producing different colors, including blue, purple, and red (Mattioli et al., 2020; Fang, 2015; Cai et al., 2022). Anthocyanins can be classified into six derivatives: pelargonidin, peonidin, cyanidin, delphinidin, petunidin, and malvidin. Different types of anthocyanins have different colors. For instance, cyanidin and its derivatives are primarily mauve or brick red, pelargonidin and its derivatives are orange or red, and delphinidin and its derivatives are purple or blue (Liu et al., 2018; Zhuang and Manzitto-Tripp, 2022). Often, the higher the anthocyanin content, the deeper the fruit color. It was reported that abiotic stresses can influence the accumulation of anthocyanins in plants, such as high temperature, low temperature, drought, salt stress, osmotic pressure, and heavy metals (Shao et al., 2025; Naing and Kim, 2021; Martinsen et al., 2020).

The biosynthetic pathway of anthocyanins commences with the phenylpropanoid metabolic process, which necessitates the involvement of numerous pivotal enzymes encoded by the corresponding structural genes, including phenylalanine lyase (*PAL*), cinnamic acid-4-hydroxylase (*C4H*), 4-coumaroyl CoA ligase (*4CL*), and chalcone synthase (*CHS*). In addition, the following enzymes are involved in anthocyanin biosynthesis: chalcone isomerase (*CHI*), flavonoid 3-hydroxylase (*F3H*), flavonoid 3’-hydroxylase (*F3’H*), and flavonoid 3’,5’-hydroxylase (*F3’,5’H*). Dihydroflavonol 4-reductase (*DFR*), anthocyanidin synthase (*ANS*), flavonoid 3-O-glucosyltransferase (*UFGT*), and glutathione Stransferase (*GST*) (Liu et al., 2018; Wang et al., 2020a). The impact of these pivotal structural genes on anthocyanin synthesis has been substantiated in a multitude of plant species, including tomato (Heredia et al., 2015), onion (Kim et al., 2016), apricot (Ni et al., 2022), and plum (Liao et al., 2023).

Transcription factors (TFs) and plant hormones such are involved in anthocyanin metabolism. It has been postulated that MYB, bHLH, NAC, ERF, and WD-40 are associated with regulatory genes of anthocyanins (Yang et al., 2023; Zou et al., 2023; An et al., 2020). Plant hormones are a class of small-molecule compounds that regulate plant growth and development via chemical transmission signals. The synergistic effects of light and various plant hormones can regulate the synthesis of anthocyanins by regulating key structural genes related to anthocyanin metabolism (Wang et al., 2023a; Ahammed and Li, 2022). In apple, a regulator of abscisic acid (ABA) signaling, MdABI5 directly binds to the promoter of *MdbHLH3* to activate its expression and enhance its binding of MdbHLH3 to its target genes, *MdDFR* and *MdUF3GT*, ultimately inducing anthocyanin biosynthesis (An et al., 2021). Many studies have used a combined metabolomic and transcriptomic approach to elucidate the mechanisms underlying fruit trait formation and regulation of associated genes. However, the types and contents of anthocyanins in raspberry fruit during different postharvest periods are yet to be documented, and the mechanism by which raspberry fruit color develops remains unclear.

The color change of postharvest raspberry fruits is evident during storage, progressing from white to pink, red, and finally to deep red. Nevertheless, the molecular mechanisms leading to these color changes have not been explored, which limits the quality and genetic improvement of raspberry fruits. The aim of the study was to systematically investigated anthocyanin dynamics in postharvest raspberry fruit through multi-omics approaches, including identification of DEGs involved in anthocyanin compounds synthesis and metabolic profiles involved in pigment accumulation from postharvest raspberries at various color stages, as well as study on the molecular mechanisms associated with anthocyanin biogenesis and deposition in red raspberries, which would provide new insights into the color change process and regulatory network of anthocyanin biosynthesis in fruits.

## 2 Materials and methods

### 2.1 Plant materials

The ‘*Harrietez*’ cultivar raspberry fruits were collected from the raspberry planting base in Shangzhi, Harbin City, Heilongjiang province as experimental material. Raspberry plants were growing under field conditions utilizing natural sunlight and rain-fed irrigation. Raspberry fruits at the white stage were harvested and transported to the laboratory within 4 h under dark conditions, maintained at 4°C with 92% relative humidity. The samples were divided into five stages based on their color during postharvest storage. RBT-1: newly harvested white fruits; RBT-2: fruits from white to pink; RBT-3: fruits turned pink; RBT-4: fruits turned red; RBT-5: fruits turned deep red. The fruits from the five stages were immediately frozen in liquid nitrogen and then stored at -80°C for transcriptome and metabolome analyses, and three biological repeats were performed for each experiment.

### 2.2 Total anthocyanin content determination

Anthocyanin content was determined using an anthocyanin assay kit (ml095256; Shanghai Enzyme-Linked Biotechnology Co. Ltd.). Cyanidin-3-O-glucoside was used to generate a standard curve, and the results were expressed in milligrams of cyanidin-3-O-glucoside equivalents per gram of raspberry.

### 2.3 Fruit color characteristics

The difference in fruit color between two opposite points on the equatorial line was measured using a WR10QC chromometer. The parameters of the L* value represent the lightness (+: black, −: white), and a* and b* indicate chromaticity (+: red, −: green) and (+: yellow, −: blue), respectively.

### 2.4 Transcriptome sequencing and analysis

Fifteen samples from three biological replicates were collected at each stage of RBT-1 ~ RBT-5 used for transcriptome sequencing. The RNA of these samples was extracted using the ethanol precipitation method and CTAB-PBIOZOL and then dissolved in 50 μL DEPC-treated water. Subsequently, the total RNA was identified and quantified using a Qubit fluorescence quantitative instrument and a Qsep400 high-pass biological fragment analyzer. RNA-Seq libraries were then prepared. Most mRNAs in eukaryotes have structural characteristics of polyA tails. Oligo (dT) magnetic beads were used to enrich mRNA with a poly (A) tail. Purified mRNA was cleaved into small fragments at an appropriate temperature with a fragment buffer, and first-strand cDNA was generated by reverse transcription using random hexamer primers. Subsequently, dUTP was used instead of dTTP to incorporate into the second strand of the cDNA. The enzyme could not amplify the uracil-containing DNA template to achieve chain specificity, thereby synthesizing the second-strand cDNA. The purified double-stranded cDNA was repaired at the end, and a tail was added to connect it to the sequencing connector. After the connection was complete, DNA magnetic bead purification and fragment selection were performed to obtain a library of 250–350 bp inserted fragments. PCR amplification was performed, and the amplification product was purified using DNA magnetic beads and dissolved in nuclear-free water to obtain a library. Subsequently, the concentration was detected using a Qubit fluorescence quantitative analyzer, and the fragment size was detected using a Qsep400 high-throughput biological fragment analyzer. Finally, the effective concentration of the library was accurately quantified by the Q-PCR method. Using Illumina Nova 6000 (Wuhan Metware Biotechnology Corporation, Wuhan, China), different libraries were pooled according to the effective concentration and amount of data required for sequencing, and 150 bp paired-end reads were generated.

Data quality control was performed using FASTP to remove reads containing adapters. Paired reads were removed under the following conditions: when the number of N in any sequencing read exceeded 10% of the length of that read and when any sequencing read contained low-quality bases (Q ≤ 20) exceeding 50% of the length of that read. Subsequent analyses were performed using the clean reads. Gene function annotation was performed using the following databases: non-redundant protein database (NR), PFAM, Clusters of Orthologous Groups for eukaryotic whole genomes (KOG), COG, Swiss-Prot protein, KEGG, KO, and Gene Ontology (GO) (Tang et al., 2022; Cao et al., 2019). DEGs analysis was conducted using DESeq2 software (1.20.0) with screening thresholds of |log2FC| ≥ 1.0 and padj < 0.05 (Ye et al., 2023).

### 2.5 Anthocyanin-targeted metabolome analysis

The raspberry samples were freeze-dried and ground into powder. Approximately 50 mg of the powder was dissolved in 500 μL of the extract (methanol: water: hydrochloric acid = 500:500:1). The mixture was vortexed, sonicated for 5 min, and centrifuged at 13400 ×*g* and 4°C for 3 min. The supernatant was collected, the residue was re-extracted, and the supernatants were combined twice. The supernatant was filtered through a 0.22 μm microporous membrane, and the filtrate was stored in an injection vial for LC-MS/MS analysis. Data were collected using a UPLC-ESI-MS/MS system (UPLC, ExionLC™AD, https://sciex.com.cn/; MS, Applied Biosystems 6500 Triple Quadrupole, https://sciex.com.cn/). The analytical conditions were as follows, UPLC: column, WatersACQUITY BEH C18 (1.7 µm, 2.1 mm × 100 mm); solvent system, water (0.1% formic acid): methanol (0.1% formic acid); gradient program, 95:5 V/V at 0 min, 50:50 V/V at 6 min, 5:95 V/V at 12 min, hold for 2 min, 95:5 V/V at 14 min; hold for 2 min; flow rate, 0.35 mL/min; temperature, 40°C; injection volume, 2 μL.

Anthocyanins were analyzed using the Scheduled Multiple Reaction Monitoring (MRM) technique performed by Metware Biotechnology (Wuhan, China). Analyst 1.6.3 software (Sciex) was used for the data collection. All metabolite quantification was performed using Multiquant 3.0.3 software (Sciex). The anthocyanin content was detected using NetWare (http://www.metware.cn/). DAMs between groups were identified using VIP ≥ 0 and |log2FC| ≥ 1.0.

### 2.6 Integrated transcriptomics and metabolomics analyses

WGCNA was conducted to identify hub genes associated with the anthocyanin metabolic pathway. Cytoscape software (version 3.10.0) was used to visualize the corresponding association networks. Pearson correlation analysis among the DEGs and DAMs was conducted, and strong correlation conditions were |r| ≥ 0.8 and *p* < 0.05. The names of the 63 structural genes related to anthocyanin synthesis, TF, and plant hormone signaling-related genes (**Supplementary Table S1**) were mainly derived from the annotation results of the NR database. For better visualization, we numbered them according to the order of the genes, such as *RiMADS1-RiMADS5*.

### 2.7 Quantitative real-time PCR analysis

Total RNA was extracted from the five stages described above for RNA-seq library construction. The concentration and integrity of RNA were determined using a NanoDrop spectrophotometer and gel electrophoresis. cDNA was synthesized using a HiScript III RT SuperMix for qPCR (+gDNA wiper) kit (R323-01; Vazyme, China). Quantitative reverse transcription polymerase chain reaction (RT-qPCR) was performed using ChamQ Universal SYBR qPCR Master Mix (Q711-02, Vazyme, China). RT-qPCR primers (**Supplementary Table S2**) were designed using Primer6.0. *Rubus idaeus 18S ribosomal RNA* (*Ri18S*) (GenBank, KP125886) was used as the internal reference gene with the following primers: *Ri18S-F*, 5`-CTACCTATTGTAAGGAATGGTGCCT-3` and *Ri18S-R*, 5`-TTCTGCATCCGAGATATCAAGTAGT-3` (Fuentes et al., 2015), the relative expression of the genes was calculated using the relative quantification method (2^-ΔΔCT^).

### 2.8 Statistical analysis

Data on the anthocyanin content and fruit color data were analyzed using one-way analysis of variance (ANOVA) with SPSS 26.0 (IBM, Armonk, NY, USA) software. The dates were expressed as the means ± SD. Statistically significant differences were assessed using the least significant difference test, with the significance set at *p* < 0.05.

## 3 Results

### 3.1 Differences of color and total anthocyanin in postharvest raspberry fruit at different color period

**Figure 1:**
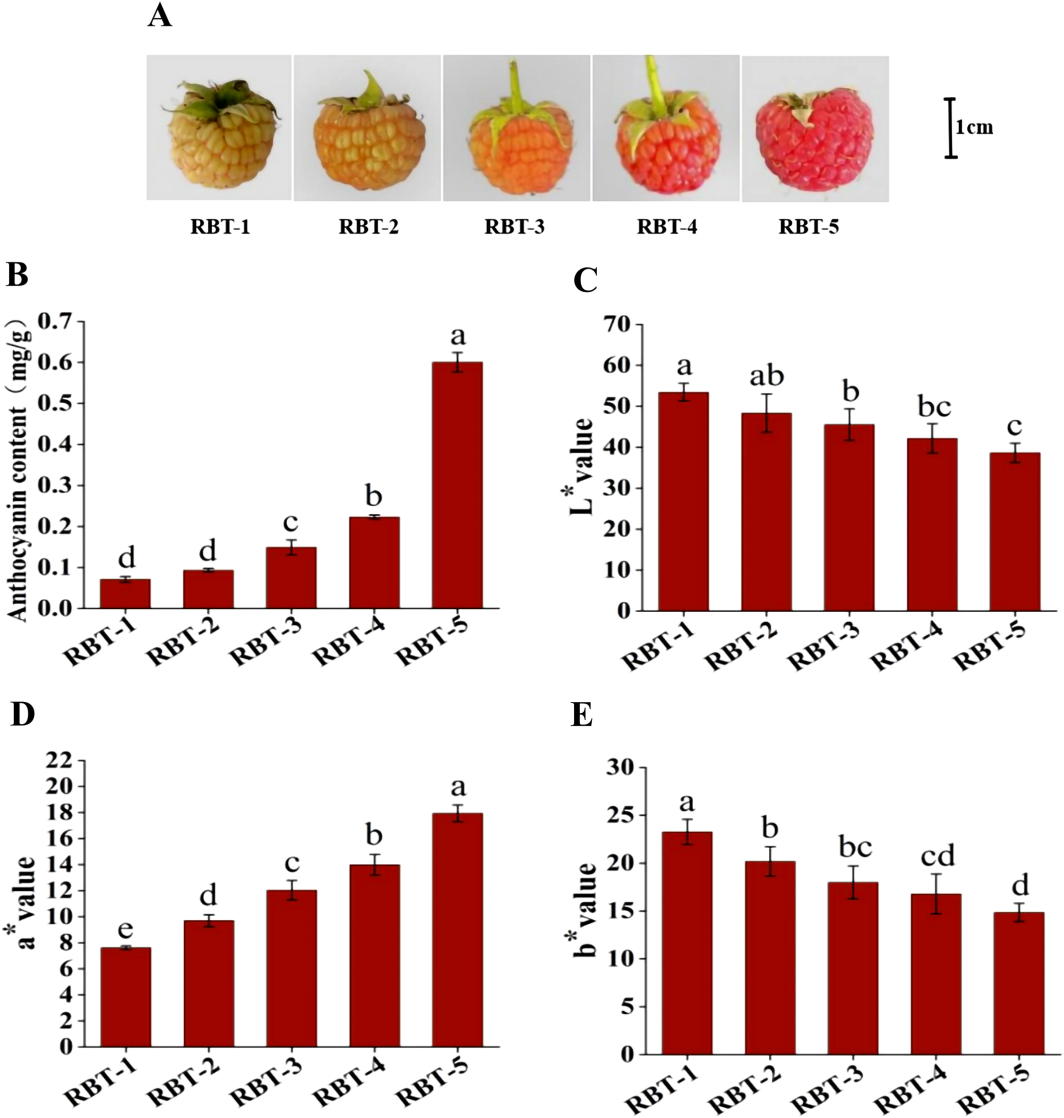
Color and anthocyanin content of raspberry fruit. **(A)** Color variation of postharvest raspberry fruit during storage. RBT-1 to RBT-5: white to deep red fruits. **(B)** Total anthocyanin content in RBT-1 and RBT-5. **(C)** L* values in the RBT-1 to RBT-5 samples. **(D)** a* values in the RBT-1 to RBT-5 samples. **(C–E)** b* values in the RBT-1 to RBT-5 samples. Values represent the mean ± standard deviation (SD) of three biological replicates. The bar chart with different lowercase letters indicates that the difference is significant (*p* < 0.05).

Following the storage of postharvest raspberries, the fruits exhibited a gradual change in color, becoming increasingly red (**Figure 1A**). Red pigmentation observed in fruits primarily comprises anthocyanins. Consequently, the total anthocyanin content of the raspberry fruit during the five color phases was quantified. Anthocyanin content was positively correlated with the degree of coloration (**Figure 1B**). The anthocyanin content of RBT-5 reached 0.6 mg/g, which was significantly higher than RBT-1, RBT-2, RBT-3, and RBT-4, at 8.45-, 6.38-, 4.03-, and 2.69-fold higher, respectively. The assessment of color differences is a common method for evaluating color changes in fruits. The L* value is used to quantify the degree of brightness. A lower L* value indicates a darker color and a lower sample brightness. The L* value of the raspberries gradually declined from RBT-1 to RBT-5, indicating a concomitant reduction in brightness (**Figure 1C**). The a* and b* values were used to quantify the degree of red and green color and the degree of yellow and blue, respectively. The a* value of the raspberry fruit gradually increased from RBT1 to RBT5, indicating a corresponding change in color from green to red (**Figure 1D**). However, the b* value exhibited a gradual decline, indicating a concomitant decrease in the green color of the fruit. This was caused by the raspberries gradually turning red (**Figure 1E**). These findings indicated that the anthocyanin concentration increased with the reddish hue observed in postharvest raspberry fruit, suggesting that anthocyanins considerable influence fruit coloration. Nevertheless, further investigation is required to determine the specific types and content of anthocyanins.

### 3.2 Metabolic differences of raspberry fruit in different color periods

**Figure 2:**
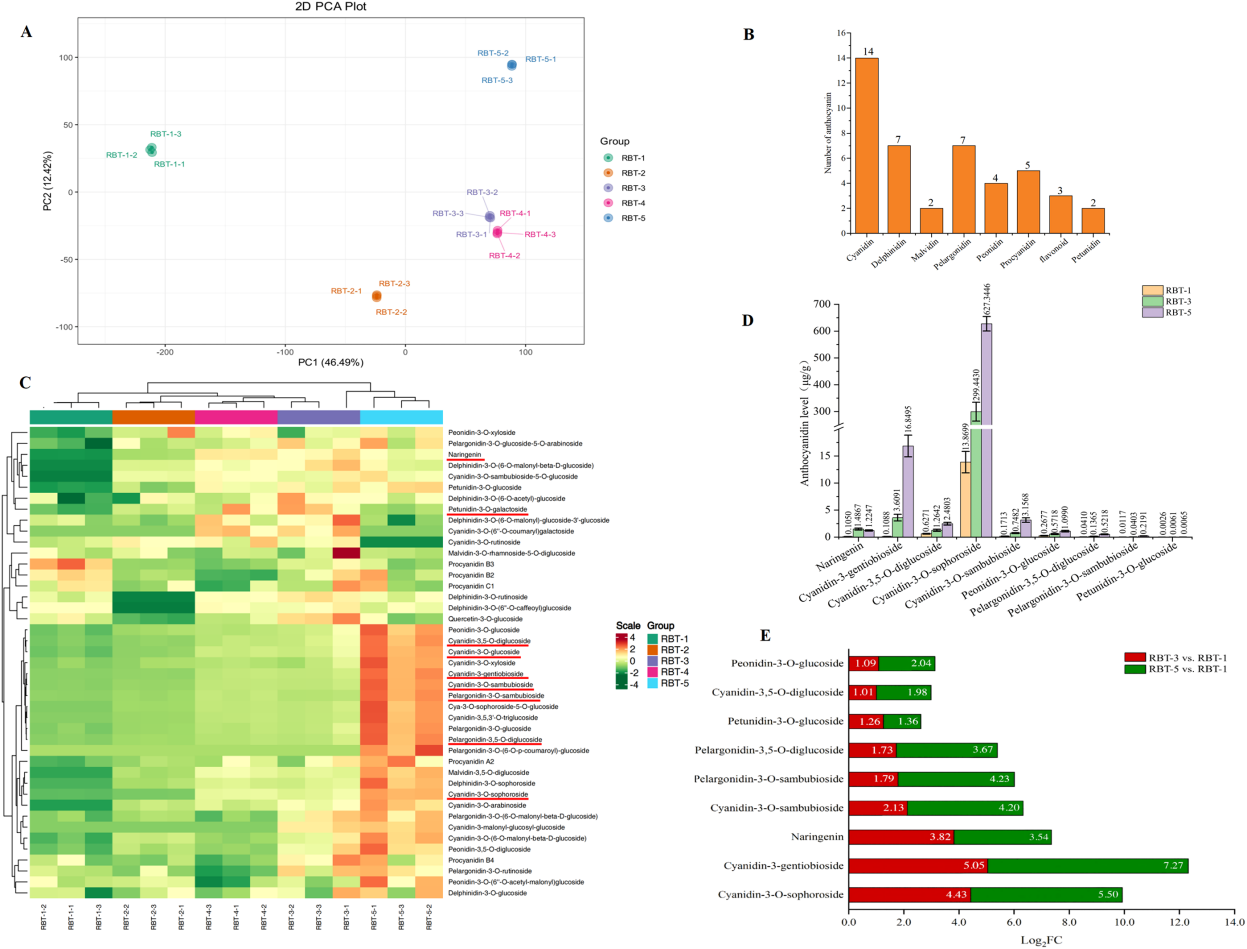
Analysis of metabolome. **(A)** PCA score plots of raspberries at different color stages. The RBT-1–1 to RBT-1-3, RBT-2–1 to RBT-2-3, RBT-3–1 to RBT-3-3, RBT-4–1 to RBT-4-3, and RBT-5–1 to RBT-5–3 represents three biological replicates for five raspberry color stages. **(B)** Anthocyanin quantity. **(C)** Heat maps of the 43 anthocyanin metabolites. **(D)** The content of 9 DAMs in RBT-1, RBT-3 and RBT-5. **(E)** log2FC values of the nine DAMs in the two comparison groups. Red represents the RBT-3 vs. RBT-1 comparison group, and green represents the RBT-5 vs. RBT-1 comparison group.

A targeted metabolomic analysis of anthocyanins was conducted on postharvest raspberries at five color stages during storage to gain further insight into the metabolites associated with red coloration in raspberries. The results of the principal component analysis (PCA) for the metabolome are presented in **Figure 2A**. The first two principal components distinguished 15 samples, accounting for 54.79% and 15.69% of the total variation, respectively. Subsequently, the samples were divided into five groups, namely RBT-1, RBT-2, RBT-3, RBT-4, RBT-5. The repeated samples within each group exhibited a high degree of correlation, whereas the distance between the components was considerable, thereby attesting to the veracity of the metabolomic data. As shown in **Figure 2B**, 43 metabolites were identified, which were classified into 8 derivatives, including 14 cyanidins, 7 delphinidins, 2 flavonoids, 2 malvidin pigments, 7 pelargonidins, 4 peonidins, 2 petunidins and 5 proanthocyanidins, with cyanidin being the most prevalent. The differentially accumulated flavonoids were subjected to further analysis to investigate the color changes observed in postharvest raspberries. The hierarchical clustering heat map was drawn by using the FPKM values of 43 metabolites. The concentrations of cyanidin-3-gentiobioside, peonidin-3-O-glucoside, pelargonidin-3,5-O-diglucoside, cyanidin-3-O-gentiobioside, cyanidin-3,5,3’-O-triglucoside, cyanidin-3-O-glucoside, and so on exhibited a continuously increasing trend and the value is constantly rising (**Figure 2C**). Nine DAMs were identified when comparing RBT-3 with RBT-1 and RBT-5 with RBT-1. All these DAMs were upregulated in both groups. The identified DAMs included four cyanidins, two pelargonidins, one peonidin, one petunidin, and one naringenin (**Figure 2D**). Among the nine DAMs, log2FC values were all positive. Only naringenin exhibited a downward trend in the log2FC value in the two comparison groups, whereas the remaining eight DAMs demonstrated an upward trend (**Figure 2E**). The cyanidin-3-O-sophoroside content was the highest among the nine DAMs, followed by cyanidin-3-gentiobioside, pelargonidin-3,5-O-diglucoside, cyanidin-3-O-sambubioside, and pelargonidin-3-O-sambubioside. This suggests that these DAMs may be the primary anthocyanins responsible for the red coloration of postharvest raspberry fruits.

### 3.3 Transcriptome analysis and differential expressed gene identification of raspberry fruit at different color stages

**Figure 3:**
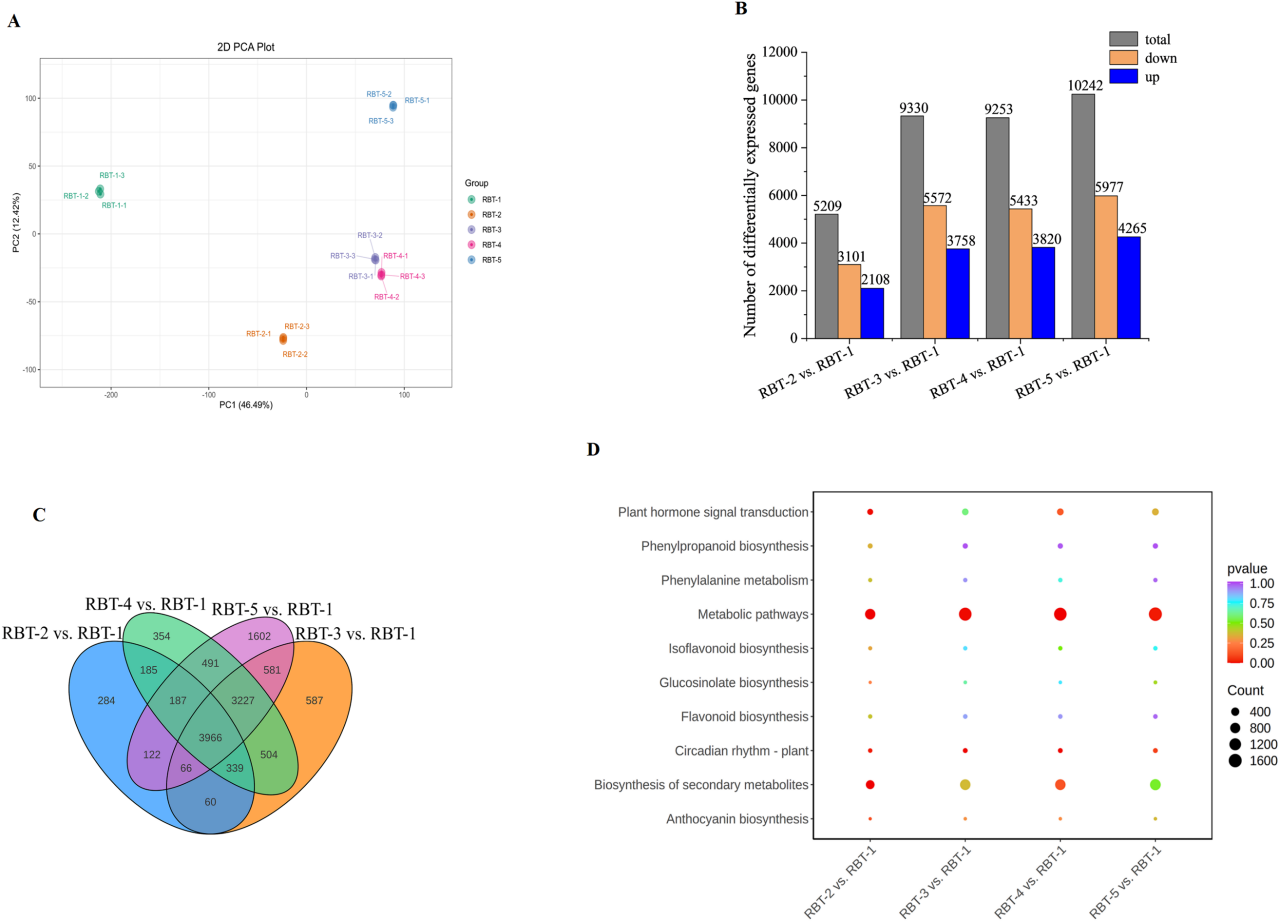
Analysis of differentially expressed genes in the transcriptome. **(A)** PCA score plot of 15 transcriptome samples. **(B)** DEGs quantity in the four groups. The thresholds is |log2FC| ≥ 1.0 and padj < 0.05. **(C)** Venn plot of DEGs at different color stages. **(D)** KEGG enrichment analysis of four groups. The q values range from 0-1. The smaller the value, the more significant the enrichment.

To elucidate the molecular mechanisms underlying anthocyanin biosynthesis in raspberry fruit, high-throughput RNA-seq analysis was conducted on 15 cDNA libraries. PCA was conducted on the comparison group, whereby the first two principal components (PC1 and PC2) accounted for 46.49% and 12.42% of the observed characteristics across the five periods, respectively. A substantial separation along the PC1 and PC2 axes was observed for RBT-1, RBT-2, RBT-3, RBT-4, and RBT-5, indicating a notable difference among the groups (**Figure 3A**). The clean reads ranged from 39,543,024 to 45,551,156, with a Q30 value exceeding 93% for each library and an average GC content of 46.9% (**Supplementary Table S3**). A total of 13,239 differentially expressed genes (DEGs) were identified in the (Non-Redundant Protein Database) NR, (Nucleotide Sequence Database) NT, Protein Family (PFAM), (Clusters of orthologous groups for eukaryotic complete genomes) KOG/Clusters of Orthologous Groups (COG), Swiss-Prot, Kyoto Encyclopedia of Genes and Genomes (KEGG) Or thology (KO), Gene Ontology (GO), and other databases through gene annotation (**Supplementary Table S4**). The DEGs of the four comparison groups were identified using the thresholds of |log2FC| ≥ 1.0 and padj < 0.05. As fruit redness increased, the number of DEGs also increased. In the RBT-2 vs. RBT-1 comparison, 5,209 DEGs were identified, of which 2,108 were upregulated and 3–101 were downregulated. In the RBT-3 vs. RBT-1 comparison, 3,758 DEGs were upregulated, and 5,572 DEGs were downregulated. In the RBT-4 vs. RBT-1 comparison, 9,253 DEGs were detected, of which 3,820 DEGs were upregulated, and 5,433 were downregulated. Finally, in the RBT-5 vs. RBT-1 comparison, 10,242 DEGs were detected, of which 4,265 were upregulated and 5,977 were downregulated (**Figure 3B**). The common or unique DEGs among the four comparison groups were analyzed, and a Venn diagram demonstrated that 3,966 DEGs were co-expressed in all four comparisons. The majority of DEGs (1,602) were in RBT-5 vs. RBT-1 (**Figure 3C**), indicating these DEGs may be responsible for the color difference among the groups.

To identify DEGs involved in metabolic pathways at different stages and to further explore the function of these genes, KEGG enrichment analyses were conducted using DEGs across the four comparison groups. Each comparison group involved 144, 145, 146, and 146 metabolic pathways, respectively (**Figure 3D**; **Supplementary Tables S5–8**). Of these, six were associated with anthocyanin synthesis: plant hormone signal transduction (ko04075), phenylpropanoid synthesis (ko00940), phenylalanine metabolism (ko00360), flavonoid biosynthesis (ko00943), flavonoid and flavonol biosynthesis (ko00944), and the plant circadian rhythm (ko04712). Plant hormone signal transduction and anthocyanin biosynthesis were significantly enriched in the RBT-5 vs. RBT-1 comparison (**Figure 3D**).

### 3.4 Analysis of key structural genes associated with anthocyanin biosynthesis pathway

**Figure 4:**
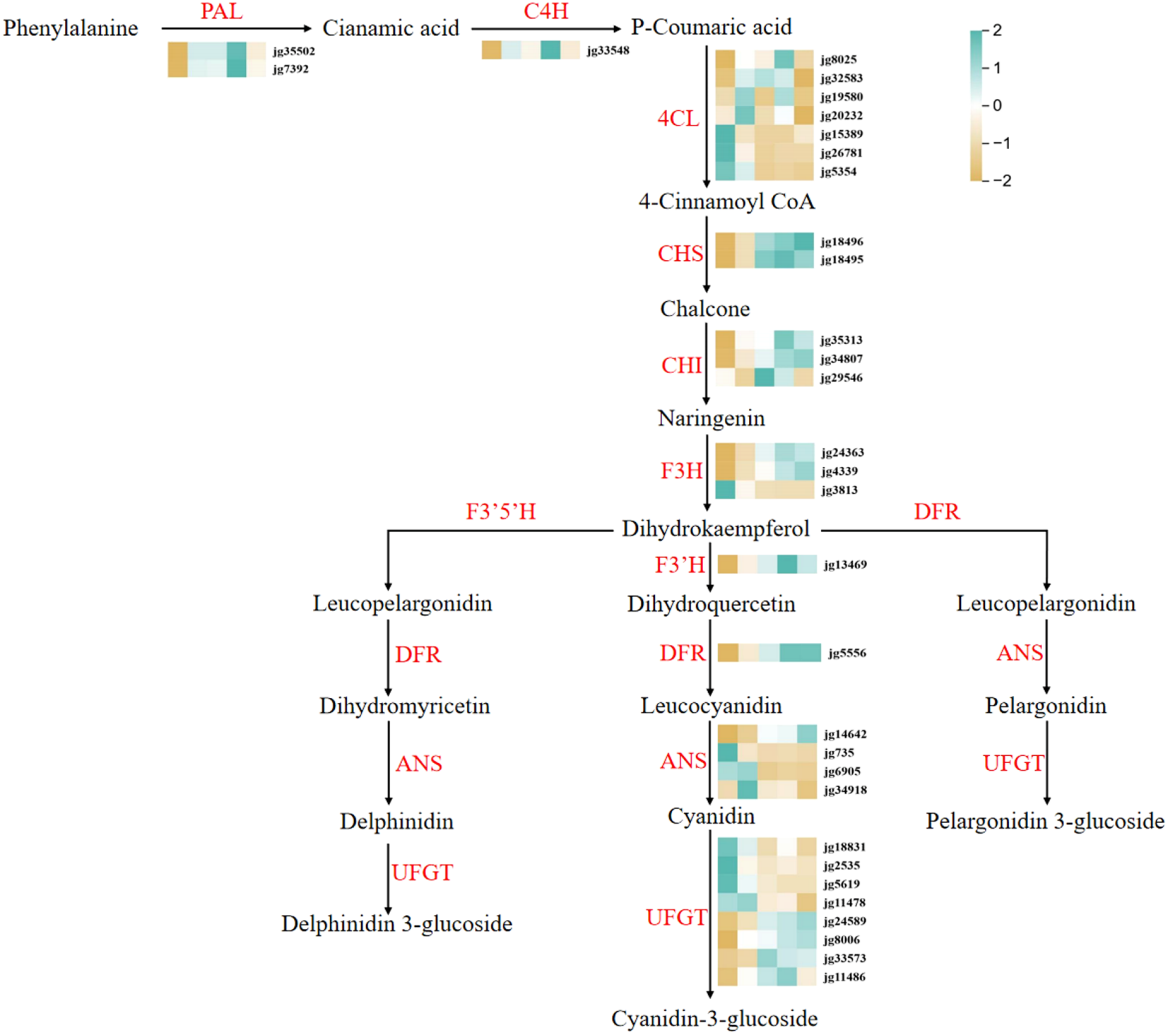
Differentially expressed structural gene regulatory network of the anthocyanin biosynthetic pathway in raspberry. Expression levels from low to high are represented as brown to blue. PAL, phenylalanine ammonia lyase; C4H, cinnamate 4-hydroxylase; CHS, chalcone synthase; CHI, chalcone isomerase; F3H, flavanone 3-hydroxylase; F3’H, flavonoid 3’-hydroxylase; DFR, dihydroflavonol 4-reductase; ANS, anthocyanin synthase; UFGT, flavonoid 3-O-glucosyltransferase.

To investigate the anthocyanin biosynthesis pathway during the postharvest color conversion of raspberry fruit, DEGs related to anthocyanin biosynthesis were identified in the transcriptome data. A total of 32 differentially expressed structural genes were identified (**Figure 4**), including one *C4H*, *F3’H* and *DFR*, two *PAL* and *CHS*, three *CHI* and *F3H*, four *ANS*, eight *4CL* and *UFGT* genes. Of these, one *C4H*, *F3’H*, *DFR* and *ANS*, two *PAL*, *CHS* and *F3H*, three *CHI*, and four *UFGT* exhibited a notable increase in expression from white to deep red fruit, whereas one *F3H*, two *ANS*, three *4CL*, and four *UFGT* were upregulated during this process. The expression pattern of these structural genes was found to be associated with changes in anthocyanin metabolism, particularly cyanidins. This suggests that these genes may regulate the anthocyanin content, which ultimately drives the white to deep red color change observed in postharvest raspberries.

### 3.5 Identification of differentially expressed TFs related to anthocyanin biosynthesis

**Figure 5:**
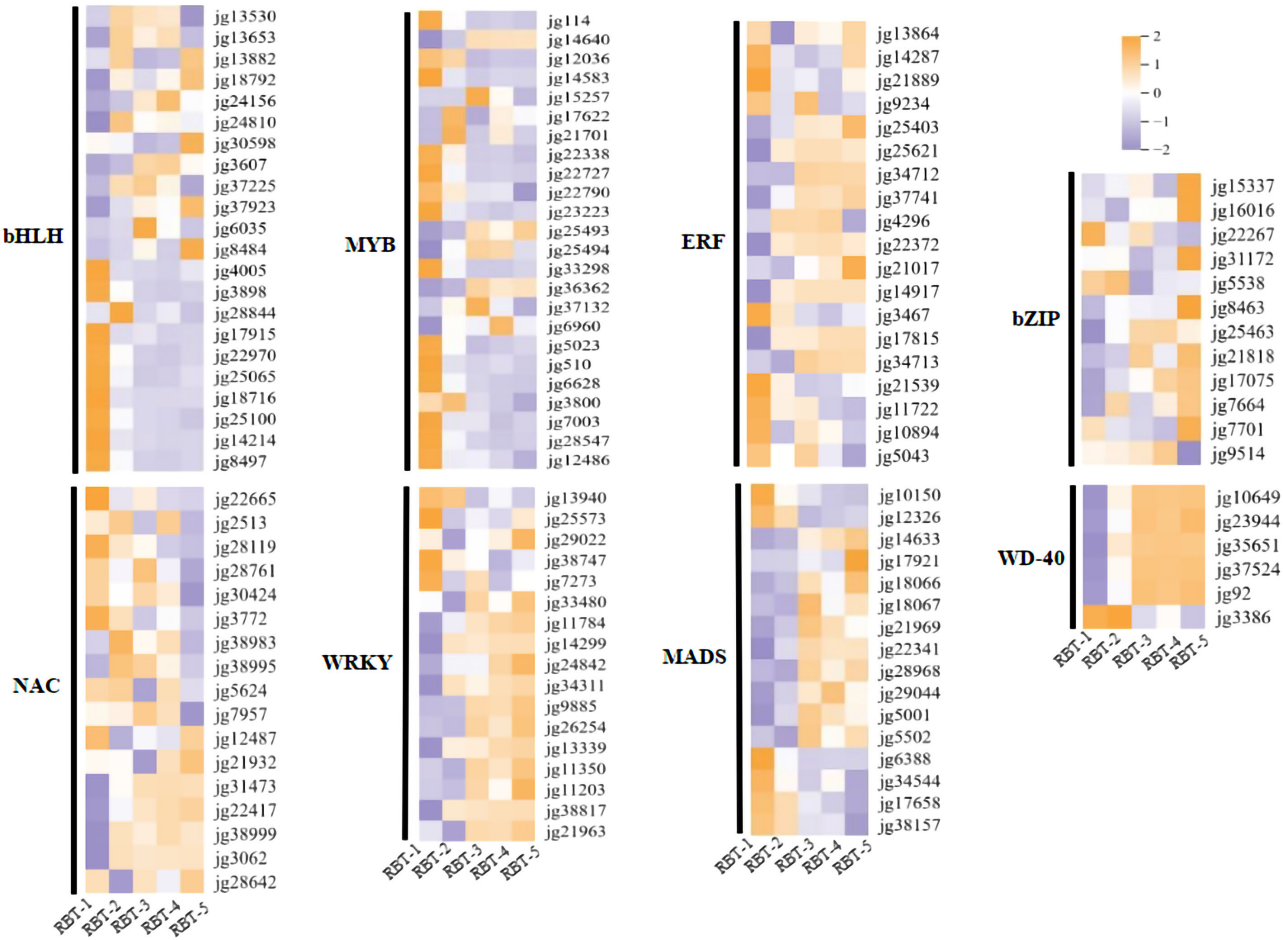
Heatmaps of differentially expressed TFs associated with anthocyanin biosynthesis pathways. Expression levels from low to high are represented as purple to orange.

MYB, bHLH, WD-40, WRKY, ERF, MADS, bZIP, and NAC TFs play a pivotal role in anthocyanin biosynthesis by regulating the expression of key structural genes. In this study, a considerable number of DEGs were TFs, including 24 MYB, 22 bHLH, 5 WD-40, 16 WRKY, 15 ERF, 16 MADS, 12 bZIP, and 16 NAC TFs (**Figure 5**). The expression heatmap indicated that the majority of bHLH, bZIP, MADS, and NAC TFs exhibited increased expression levels. Most MYB, WD-40, WRKY, and ERF TFs exhibited downward regulation. The expression levels of specific TFs, including all WD-40 TFs (*jg10649*, *jg23944*, *jg35651*, *jg37524*, *jg92*, and *jg3386*), 12 MYB TFs (*jg114*, *jg14583*, *jg22338*, *jg22727*, *jg23223*, and *jg7003*), and nine bHLH TFs (*jg4005*, *jg3898*, *jg25065*, *jg18716*, and *jg8497*), were significantly altered. The expression levels of six MADS, six WRKY, and four NAC TFs were significantly higher in stage 1 (RBT-1) than in stages 2, 3, 4, and 5. This indicates a negative correlation with anthocyanin biosynthesis. However, the majority of TFs exhibited a positive correlation with anthocyanin biosynthesis, including *jg36362* (MYB), *jg8484* (bHLH), *jg22417* (NAC), and *jg17921* (MADS), although the expression peaks of these TFs were observed in different samples. These findings suggest that TFs may play a pivotal role in the promotion of anthocyanin biosynthesis by regulating the expression of essential structural genes involved in anthocyanin metabolism.

### 3.6 Analysis of DEGs related to plant hormone pathway among colored raspberry

**Figure 6:**
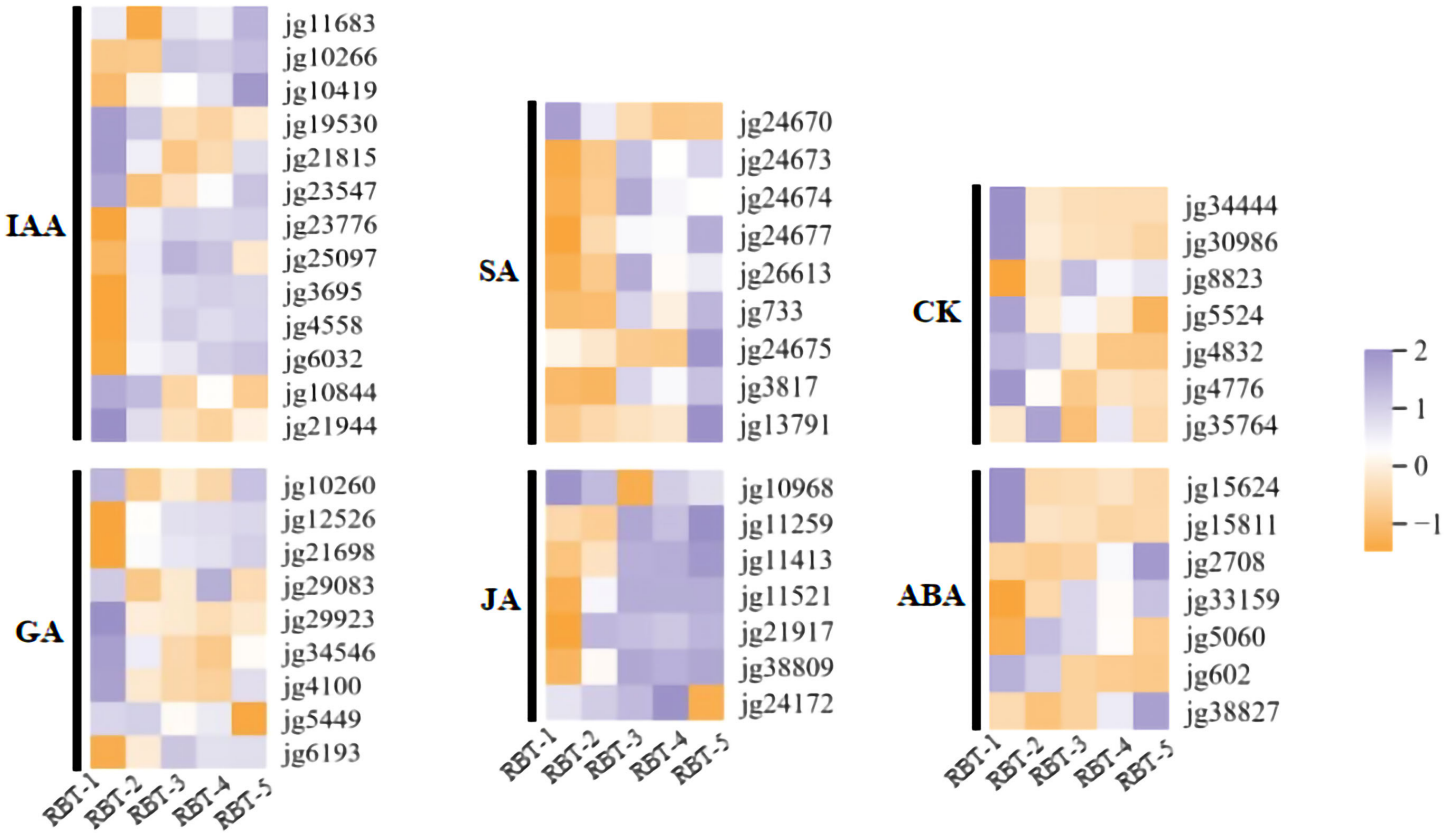
Heatmaps of DEGs participating in plant hormone metabolism. Expression levels from low to high are represented as orange to purple.

Plant hormones, including ABA, cytokinin (CK), auxin (IAA), salicylic acid (SA), jasmonic acid (JA), and gibberellin (GA), perform a variety of functions that are essential for fruit development, growth, and maturation. A transcriptome study of postharvest raspberries identified multiple DEGs associated with plant hormone signal transduction. The expression heatmaps demonstrated that nine genes were components of the GA signaling pathway, with the majority exhibiting increased expression at the RBT-2, RBT-3, and RBT-4 stages. Conversely, a few of these genes displayed a downward trend in color variation (**Figure 6**). Of these, *jg21698*, *jg29083*, and *jg29923* were identified as gibberellin 2-dioxygenases, and the observed trends in their expression differed. Furthermore, three ABA signal transduction genes exhibited decreased expression levels, whereas four genes demonstrated increased expression levels with deepening the red color in postharvest raspberries during storage. For instance, *jg15811* and *jg5060* were identified as ABA 8’-hydroxylase *CYP707A2* and ABA 8’-hydroxylase *CYP707A1*, respectively. However, the two genes exhibited contrasting expression patterns, suggesting that the equilibrium between ABA biosynthesis and catabolism is rigorously regulated during the color transition of raspberry fruit. Furthermore, CK pathway genes annotated as cytokinin hydroxylase (*jg34444*), cytokinin nucleoside 5’-monophosphate ribose phosphate hydrolase (*jg30986*), and cytokinin dehydrogenase (*jg5524*) exhibited decreased expression during color changes. The expression of most SA-binding proteins (*jg24670*, *jg24673*, *jg24674*, *jg24677*, *jg26613*, and *jg24675*) was upregulated, with only one gene (*jg24670*) exhibiting the opposite trend. This suggests that these genes may play a role in the postharvest color change of raspberries by regulating the biosynthesis of plant hormones.

### 3.7 Weighed gene co-expression network analysis

**Figure 7:**
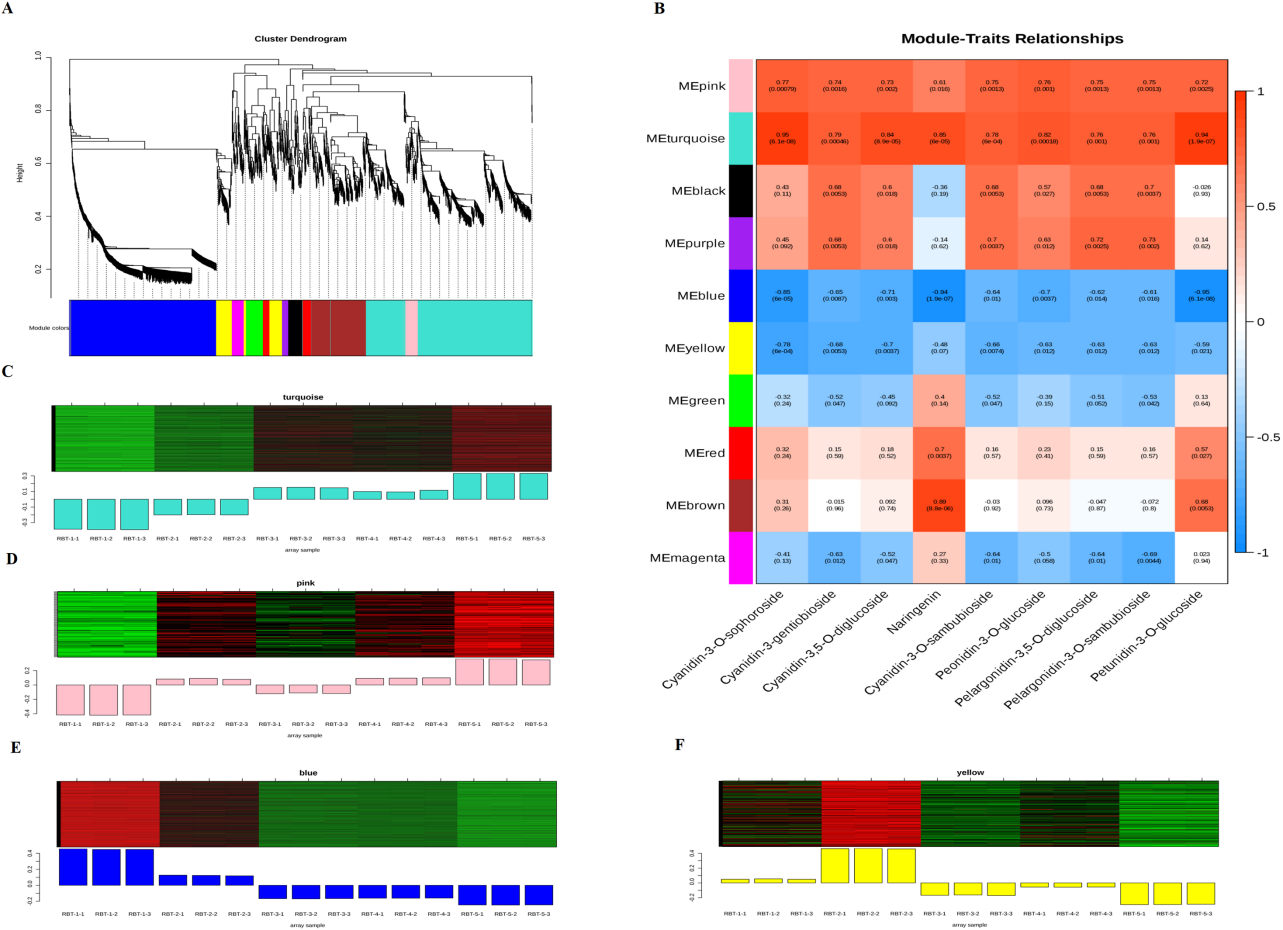
Weighted gene co-expression network analysis. **(A)** Cluster tree diagram of DEGs identified by WGCNA. **(B)** Heatmap of the correlation between DAMs and modules. **(C)** Expression trends of DEGs in the turquoise module. **(D)** The expression trend of DEGs in pink module. **(E)** Expression trends of DEGs in the blue module. **(F)** Expression trends of DEGs in the yellow module.

To investigate the relationships between DEGs and nine major anthocyanins and to explore the molecular mechanism of postharvest raspberry fruit color change during storage, we conducted a weighted gene co-expression network analysis (WGCNA) using 13–239 DEGs with a soft threshold standard of > 0.8, a soft threshold set to 10, a mergeCutHeight of 0.2, and a minimum module size of 30. This resulted in the screening and classification of 2–648 DEGs into 10 modules, each containing highly related and co-expressed DEGs (**Figure 7A**; **Supplementary Table S9**). The turquoise module exhibited the highest number of DEGs, with 882, whereas the purple module demonstrated the lowest number, with only 36. The correlation coefficients between the modules and the nine DAM contents exhibited a range from -0.95 to 0.95 (**Figure 7B**). It was observed that the contents of cyanidin-3-O-sophoroside, cyanidin-3-O-gentiobioside, and cyanidin-3-O-sambobioside exhibited the highest positive correlations with the turquoise and pink modules, and the highest negative correlations with the blue and yellow modules. The heatmap of the turquoise and pink modules demonstrated that the expression levels of these DEGs exhibited upregulated with fruit color variation (**Figures 7C, D**). Conversely, the blue and yellow modules exhibited downward trends during this process (**Figures 7E, F**). KEGG enrichment analysis revealed that the DEGs of the turquoise and blue modules were significantly enriched in metabolic pathways, biosynthesis of secondary metabolites, and ribosomes (**Supplementary Figures S1, S2**). In contrast, the pink and yellow modules were enriched in plant hormone signal transduction, anthocyanin biosynthesis, phenylpropanoid biosynthesis, and phenylalanine, tyrosine, and tryptophan biosynthesis (**Supplementary Figures S3, S4**). These findings indicate that the DEGs in the turquoise, blue, pink, and yellow modules are integral to the accumulation of anthocyanins and the pigmentation of postharvest raspberries. Ten anthocyanin synthesis structural genes, 32 TFs, and 20 genes involved in plant hormone signal transduction (ko04075) were identified as candidate genes through screening and selection from the WGCNA modules (**Supplementary Table S1**).

### 3.8 Correlation network analysis

**Figure 8:**
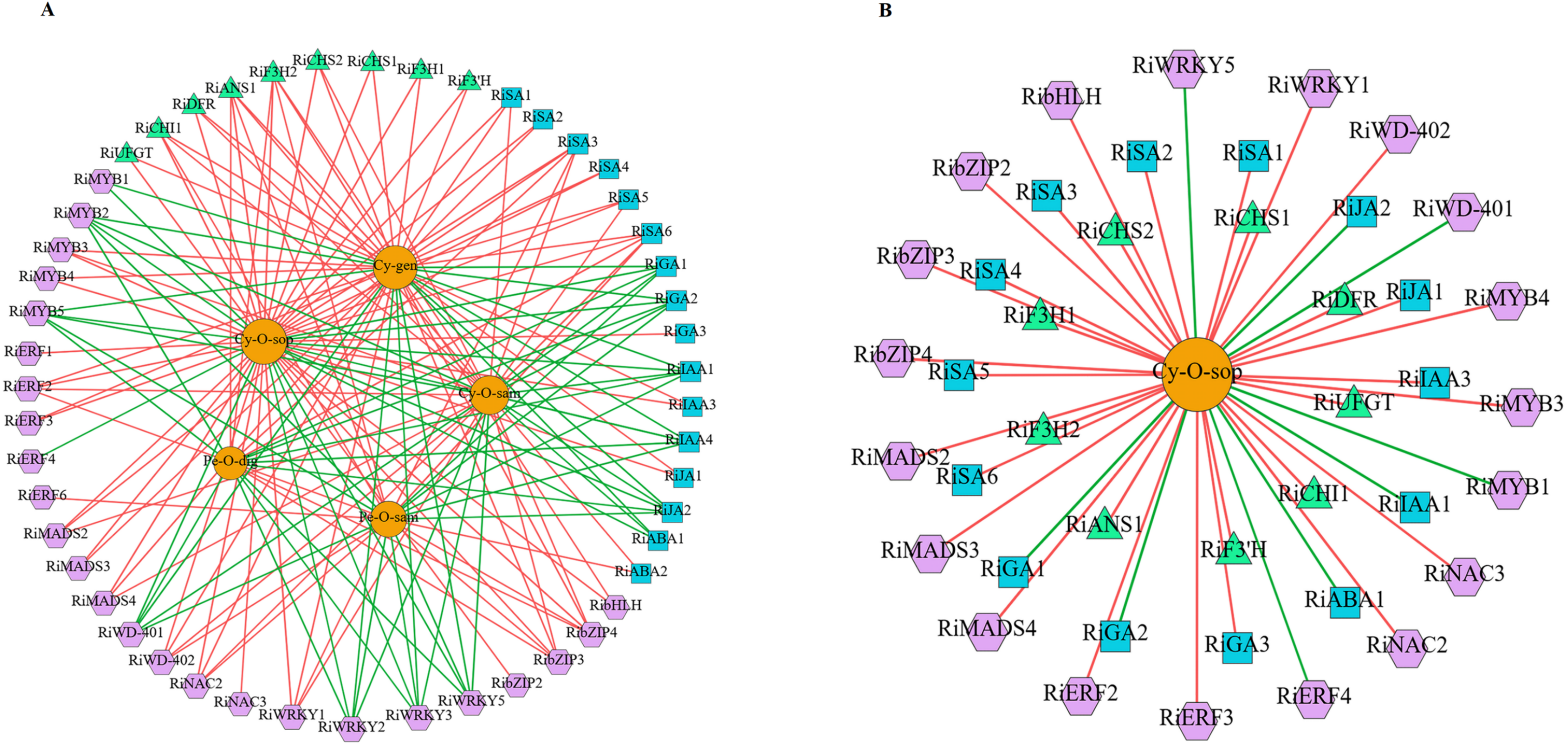
Network diagram of anthocyanin synthesis-related genes and metabolites in raspberry fruit. **(A)** Correlation network diagram of five DAMs and candidate DEGs. **(B)** Correlation network diagram of cyanidin-3-O-sophoroside and candidate DEGs. The square is described as a plant hormone signal gene, triangle is described as a structural gene, and hexagon is described as a TFs.. Cy-O-sop, Cyanidin-3-O-sophoroside; Cy-gen, Cyanidin-3-gentiobioside; Pe-O-dig, Pelargonidin-3,5-O-diglucoside; Cy-O-sam, Cyanidin-3-O-sambubioside; Pe-O-sam, Pelargonidin-3-O-sambubioside.

To further elucidate the correlation between DEGs and differentially abundant metabolites (DAMs), we constructed a relative network diagram by performing a Pearson correlation analysis on the screened genes that participated in anthocyanin synthesis and plant hormone signaling pathways. In addition, TFs and five principal anthocyanins (log2FC > 3) were identified, including cyanidin-3-O-sophoroside, cyanidin-3-gentiobioside, pelargonidin-3,5-O-diglucoside, cyanidin-3-O-sambubioside, and pelargonidin-3-O-sambubioside. The nine structural genes included one *F3’H*, *DFR*, *ANS*, *UFGT* and *CHI*, and two *F3H* and *CHS*. The green and red lines indicate positive and negative correlations, respectively. In addition, the GO enrichment analysis of these DEGs functions showed that they were significantly enriched in flavonoid biosynthetic process, flavonoid metabolic process, and transcription regulator activity (**Supplementary Figure S5**). The results demonstrated a significant correlation between the five DAMs and candidate DEGs. A total of 163 significantly correlated pairs were identified, of which 61 exhibited negative correlations, and 102 demonstrated positive correlations (**Figure 8A**; **Supplementary Table S10**). The two structural genes, *RiF3H2* and *RiANS1*, exhibited positive correlations with five metabolites, whereas *RiCHI1* demonstrated positive correlations with four metabolites, except for pelargonidin-3,5-O-diglucoside (r > 0.8 and *p* < 0.01). Two TFs, *RibZIP3* and *RibZIP4*, were positively correlated with the five DAMs, whereas six TFs, *RiMYB2*, *RiMYB5*, *RiWRKY2*, *RiWRKY3*, *RiWRKY5*, and *RiWD-401*, were negatively correlated with the five DAMs. In addition, the plant hormone signaling pathway genes *RiSA3* and *RiSA6* demonstrated a significant positive correlation with the five predominant anthocyanins, whereas the five genes *RiIAA1*, *RiIAA4*, *RiJA2*, *RiGA1*, and *RiGA2* exhibited a significant negative correlation (**Figure 8A**; **Supplementary Table S10**). Furthermore, a correlative analysis was conducted between the most abundant pigment, cyanidin-3-O-sophoroside, in the coloration of raspberry fruit and the DEGs in the turquoise, pink, blue, and yellow modules that were differentially expressed in the RBT-4 vs. RBT-1 and RBT-5 vs. RBT-1 comparison groups (log2FC > 1) (**Figure 8B**; **Supplementary Table S11**). A total of 42 DEGs were significantly correlated with cyanidin-3-O-sophoroside. Of these, 9 were structural genes, 19 were TFs, and 14 were plant hormone signaling pathway genes. All structural genes exhibited a positive correlation, whereas only four TFs (*RiMYB1*, *RiWRKY5*, *RiERF4*, and *RiWD-401*) and five hormone-related genes were negatively correlated (**Figure 8B**; **Supplementary Table S11**). The following genes were significantly correlated with cyanidin-3-O-sophoroside levels (r > 0.95, *p* < 0.01): *RiCHS1*, *RiCHS2*, *RiCHI1*, *RiF3H1*, *RiF3H2*, *RiDFR*, *RiANS1*, *RiMYB3*, *RiMADS4*, *RiWRKY1*, *RiWD-401*, *RibZIP4*, *RiNAC2*, *RiSA1*, *RiSA3*, *RiJA2*, *RiGA1*, and *RiGA2*. The correlation between *RiANS1* and cyanidin-3-O-sophoroside was the most significant, with a correlation coefficient exceeding 0.99 and a p-value less than 0.01. These findings indicate that TFs and associated plant hormones may regulate the expression of structural genes involved in anthocyanin synthesis, thereby influencing the synthesis of anthocyanins, particularly cyanidin-3-O-sophoroside.

### 3.9 Quantitative real-time PCR analysis

**Figure 9:**
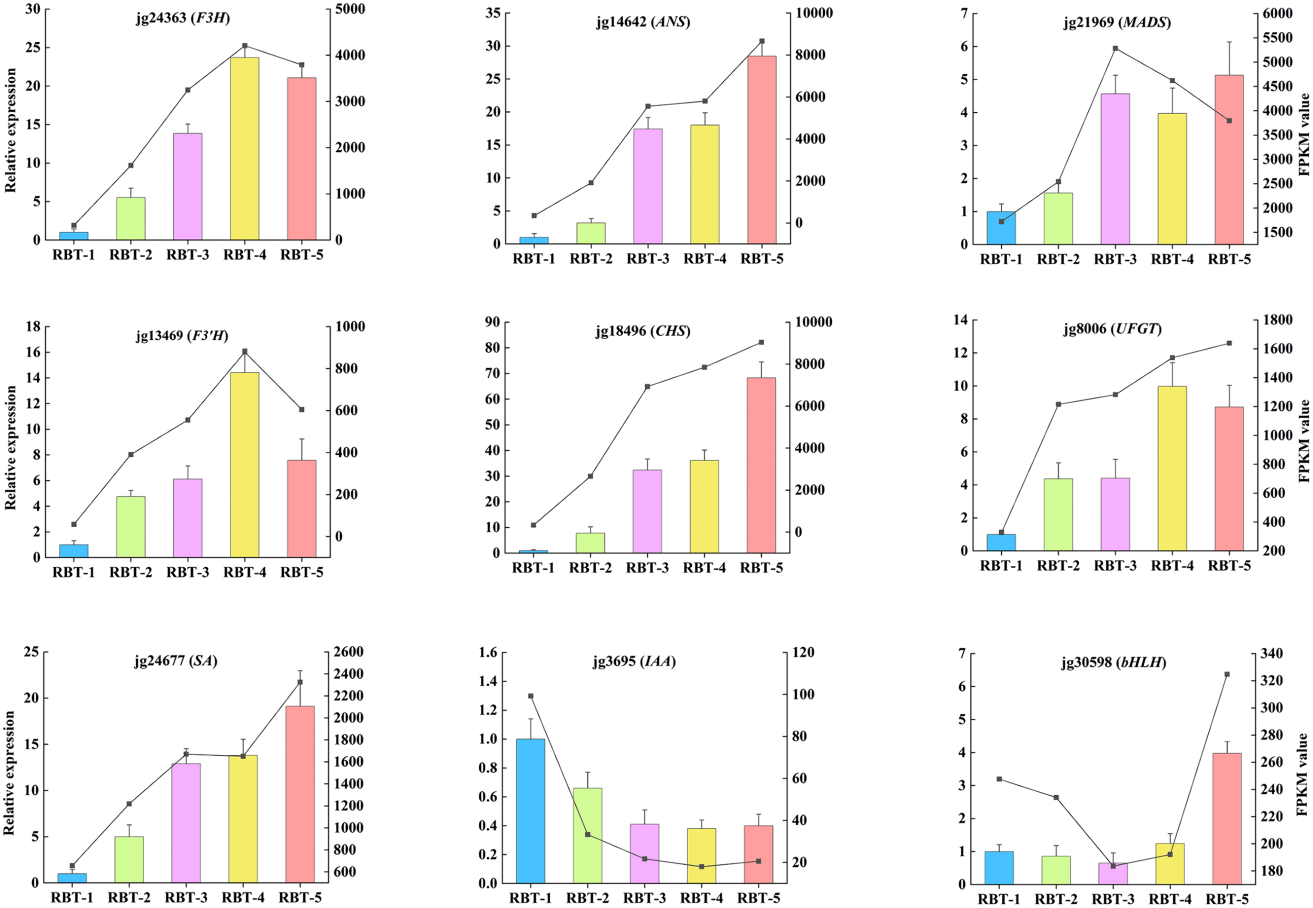
RT-qPCR validation of nine key genes related to anthocyanin content in color-variant raspberry during storage. Error bars indicate the standard deviation (SD) of three technical repeats.

To ascertain the veracity and dependability of the transcriptome sequencing data, 5 structural genes, 2 TFs and 2 hormone signaling genes implicated in the anthocyanin biosynthesis pathway were screened out (|log2FC| ≥ 1.0) for RT-qPCR analysis (**Figure 9**). According to the Pearson’s correlation coefficient (**Supplementary Table S2**), the correlation between the relative expression levels and FPKM values ranged from 0.823 (*bHLH*) to 0.994 (*F3H*). The results demonstrated that the RT-qPCR results were in accordance with those of the RNA-seq analysis, thereby substantiating the reliability and accuracy of the RNA-seq data.

## 4 Discussion

### 4.1 The positive correlation between anthocyanin accumulation and color formation

The external quality of the raspberries was significantly influenced by coloring. Anthocyanins are water-soluble pigments responsible for the red coloration of fruits (Zhao et al., 2023; Yan et al., 2021; Ji et al., 2020). Several studies have elucidated the correlation between anthocyanin accumulation and color formation in various fruit species, including *Cerasus humilis* (Ji et al., 2020), apples (Cheng et al., 2022; Li et al., 2018), and sweet cherries (Zhang et al., 2023b). Nevertheless, the mechanisms underlying anthocyanin biosynthesis in raspberries remain unclear.

To investigate the molecular mechanisms of anthocyanin accumulation and color changes in postharvest raspberries, an anthocyanin-targeted metabolome analysis was conducted. Elevated anthocyanin levels correlated with fruit coloration (**Figure 2B**), with eight derivatives identified (cyanidin, delphinidin, pelargonidin, malvidin, peonidin, petunidin, proanthocyanidin, and flavonoids) (**Figures 2C, D**). Cyanidin derivatives contribute to the formation of red-purple pigments, whereas delphinidin derivatives are responsible for the production of purple and dark colors (Khoo et al., 2017). However, the primary anthocyanins in raspberries are cyanidin-3-O-sophoroside, cyanidin-3-O-glucoside, and cyanidin-3-O-sambubioside, and their specific composition varies depending on the cultivar (Anjos et al., 2020). In freeze-dried raspberries, cyanidin-3-O-sophoroside and cyanidin-3-O-glucoside were the primary components, comprising approximately 77% and 18% of the total anthocyanin content, respectively (Marino et al., 2024a). In consistent with this study, the cyanidin derivatives detected in this study were the most abundant, and cyanidin-3-O-sophoroside was the most abundant anthocyanin in the red raspberry fruit (**Figure 2D**). Different from our results, cyanidin-3-glucoside, cyanidin chloride, and pelargonidin-3-glucoside were the most abundant anthocyanin present in dark red color strawberry fruits (Lin Y. et al., 2022). The differences may originate from distinct species-specific genetic regulatory networks and divergent evolution of anthocyanin biosynthetic pathways. Furthermore, the contents of other cyanidins, including cyanidin-3-gentiobioside and cyanidin-3-O-sambubioside, were higher in RBT-3 and RBT-5 than in RBT-1. However, despite the presence of seven delphinidin derivatives, the abundance of delphinidin was low (**Figure 2B**) by the observed color phenotype and color difference in the raspberry samples. This suggests that cyanidin-3-O-sophoroside may be the primary contributor to the color variation observed in the postharvest raspberries. In addition, pelargonidin has been documented as a red pigment in select fruits (Jaakola, 2013). In accordance with these results, seven pelargonidin derivatives were identified. The relative levels of pelargonidin-3,5-diglucoside and pelargonidin-3-O-sambubioside were 3.67- and 4.23-fold higher in RBT-5 than in RBT-1, respectively. This indicated this was also the primary reason for the color change in the raspberry fruit. These findings further suggest that anthocyanin accumulation plays a pivotal role in the pigmentation of postharvest raspberries during storage.

### 4.2 The functional contribution of structural genes to anthocyanin biosynthesis

The discrepancies in plant anthocyanin composition can be attributed to variations in gene expression along biosynthetic pathways. Given these findings, we conducted further research into the regulatory mechanisms underlying anthocyanin accumulation and coloration in raspberry fruits, focusing on the expression of metabolites and differential genes. The biosynthesis of anthocyanins occurs through two distinct pathways: the early general phenylpropanoid pathway and the late flavonoid pathway (Su et al., 2023). PAL, C4H, and 4CL are pivotal enzymes that facilitate the conversion of phenylalanine to 4-coumaroyl-CoA. CHS, CHI, F3H, F3’H, F3’5’H, DFR, ANS, and UFGT are essential enzymes that catalyze the conversion of 4-coumaroyl-CoA to synthesize anthocyanin and other flavonoid compounds (Tanaka et al., 2008). In strawberry, RNA-seq data showed that the genes of *CHS*, *CHI*, *F3H* and *DFR* were all up-regulated in red skin fruit (Xiao et al., 2024). Similarly, our RNA-seq results demonstrated that the expression levels of *RiPAL* (*jg35502* and *jg7392*), *RiC4H* (*jg33548*), *RiCHS* (*jg18496* and *jg18495*), *RiCHI* (*jg35313* and *jg34807*), and *RiF3H* (*jg24363* and *jg4339*) were higher in the red raspberry fruit than in wight raspberry fruit. The expression of *RiDFR* (*jg5556*), *RiANS* (*jg14642*), and *RiUFGT* (*jg24589*, *jg8006*, *jg33573* and *jg11486*) was elevated with the formation of red coloration in raspberry fruit (**Figure 4**).

F3H catalyzes the conversion of naringenin to dihydroflavonols, DFR reduces dihydroflavonols to leucoanthocyanins, and ANS converts them to anthocyanidins (Hichri et al., 2011). The expression of *CitF3H* increases rapidly with the accumulation of anthocyanins in blood oranges (*Citrus sinensis*) during the ripening process (Ma et al., 2023), whereas RNAi-mediated silencing and mutation of *FvF3H* impede anthocyanin biosynthesis and results in diminished anthocyanin levels, ultimately producing colorless strawberry fruit (Jiang et al., 2013). However, the downregulation of mulberry *MazsF3H* in tobacco resulted in the co-expression of *F3’H* and *ANS*, which are involved in anthocyanin biosynthesis, and a significant decrease in anthocyanin content. Similarly, overexpression of *MazsF3H* also resulted in decreased anthocyanin content (Dai et al., 2022). In this study, the expression of *RiF3H* (*jg24363* and *jg4339*) exhibited a gradual increase in correlation with the change in raspberry fruit color, reaching their highest values in RBT-4 and RBT-5, respectively. This expression was positively correlated with the changes in raspberry fruit color.

ANS is the primary enzyme involved in anthocyanin synthesis (Cai et al., 2024). The study revealed that overexpression of *SmANS* resulted in a more pronounced purple-red coloration in the leaf margins and stem segments of *S. miltiorrhiza* Bge f. alba plantlets (Li et al., 2019a). The loss of all sequences or an important second exon of *ANS* results in the emergence of white flower variation in *S. miltiorrhiza* (Lin C. et al., 2022). The study revealed that three *RiANS* (*jg735*, *jg6905*, and *jg34918*) exhibited a downward trend during the color variation process in postharvest raspberries during storage (**Figure 4**). Only *jg14642* showed a similar trend with the increase in anthocyanin content (**Figure 4**; **Figure 1B**). The correlation network diagram demonstrated that *ANS* was positively correlated with the synthesis of the five major anthocyanins (**Figure 8A**; **Supplementary Table S10**), indicating that ANS plays a pivotal role in anthocyanin biosynthesis. Glycosylation is a crucial step in the conversion of unstable anthocyanins into stable anthocyanins, as well as in the transport of anthocyanins into vacuoles, ultimately helping to accumulate plant anthocyanins.

The role of UFGT in this process is significant (Cheng et al., 2014). The results demonstrated a positive correlation between the accumulation of anthocyanin metabolites and the expression of specific *UFGTs* during the red-turning process in the raspberry fruit. The expression of *RiUFGT* (*jg8006*, *jg24589*, and *jg33573*), which is involved in anthocyanin accumulation, gradually increased during the color change in raspberry fruit (**Figure 4**). Among these, *jg8006* exhibited a positive correlation with cyanidin-3-O-sophoroside and cyanidin-3-gentiobioside (r > 0.85, *p* < 0.01) (**Supplementary Table S10**). Furthermore, it has been demonstrated that the activity of UFGT increases during the process of fruit development (Ji et al., 2021). Thus, it can be postulated that *RiUFGT* plays a significant role in the accumulation of anthocyanins during the color variation observed in raspberry fruit.

### 4.3 Transcription factors modulate anthocyanin biosynthesis through regulating target genes

The expression of key structural genes involved in anthocyanin biosynthesis is regulated by multiple TFs, including MYB, WRKY, NAC, and bHLH (Jaakola, 2013; Han et al., 2023; Jiang et al., 2017; Jin et al., 2016). The MBW protein complex, comprising MYB, bHLH, and WDR, plays a pivotal role in the regulation of anthocyanin synthesis (Xu et al., 2015; Dubos et al., 2010). MYB TFs are core regulators of MBW components. They represent one of the largest families of TFs in plants and exhibit complex functional differentiations. In kiwifruit, the cMYBF110-AcbHLH1-AcWDR1 complex has been demonstrated to directly target the promoters of anthocyanin biosynthetic genes, including *AcCHS*, *AcF3’H*, *AcANS*, *AcUFGT3a*, and *AcUFGT6b*, thereby activating their expression (Liu et al., 2021). A recent report indicated that the MBW complex CaAN1-CaGL3-CaTTG1 affects pigment accumulation and male gametophyte development in a Cha1 pepper mutant (Wang et al., 2023b). MYB61 has been demonstrated to negatively regulate anthocyanin accumulation in the petals of *Paphiopedilum phyllanthi* by regulating the *F3H* and *CHS* genes, resulting in the whitening of petals (Li et al., 2021). The involvement of DoMYB5 and DobHLH24 in the regulation of anthocyanin accumulation in *Dendrobium officinale* has been previously demonstrated (Yang et al., 2023). The findings of this study align with those of previous studies in this field. In raspberry fruits, the expression of *RiMYB1* (*jg6628*) showed a declining trend during the process of color change (**Figure 5**), which was contrary to the alteration in anthocyanin content. In contrast, a positive correlation was observed between *RiMYB5* (*jg25493*) and anthocyanin content during fruit color change, suggesting that *RiMYB1* (*jg6628*) and *RiMYB5* (*jg25493*) may play opposing roles in anthocyanin metabolism in raspberry fruit. However, this hypothesis requires further experimental validation.

bHLH TFs have been demonstrated to independently regulate the expression of *CHS*, *DFR*, and *UFGT*, thereby modulating anthocyanin biosynthesis in mulberry fruit (Li et al., 2020). In addition, SmbHLH60 and SmMYC2 have been demonstrated to exert antagonistic effects on anthocyanin biosynthesis in *Salvia miltiorrhiza* (Liu et al., 2022). Moreover, the MdMYB305-MdbHLH33-MdMYB10 complex has been shown to regulate anthocyanin balance in red-fleshed apples (Zhang et al., 2023a). In strawberry, some of bHLH and WD-40 were both upregulated or down-regulated in red skin fruit (Xiao et al., 2024). Similarly, this study observed significant upregulation for *RiMYB3* (*jg36362*) and *RibHLH* (*jg3607*) during raspberry color variation during storage. In contrast, *RiMYB2* (*jg22790*), *RiMYB5* (*jg3800*), *RiWD-40* (*jg10649*, *jg23944*, *jg35651*, *jg37524*, and *jg92*) and nine *bHLH* (*jg4005*, *jg3898*, *jg17915*, *jg22970*, *jg25065*, *jg18716*, *jg25100*, *jg14214*, and *jg8497*) exhibited opposed trends (**Figure 5**). Correlation network analysis revealed a significant positive correlation between *RiMYB3* and *RibHLH* (*jg3607*), and the predominant cyanidin-3-O-sophoroside and cyanidin-3-gentiobioside anthocyanins. In addition, *RiMYB3* was strongly correlated with cyanidin-3-O-sambubioside (r > 0.8 and *p* < 0.01), indicating that the accumulation of cyanidin-3-O-sambubioside might be regulated by *RiMYB3. RiMYB2*, *RiMYB5*, and *RiWD-401* (*jg92*) exhibited a significant negative correlation with the five DAMs (**Figure 8**; **Supplementary Table S10**), suggesting that MYB, bHLH, and WD-40 TFs may form a complex that positively or negatively regulates structural gene expression, thereby mediating anthocyanin accumulation and influencing raspberry fruit coloration. Therefore, the relationships across these TFs and anthocyanin accumulation are worthy of further study.

In pepper, co-expression regulatory networks have indicated that *CHS*, *DFR*, *CHI*, and two *WRKY44* genes are involved in anthocyanin synthesis (Zhou et al., 2022). The TFs WRKY44 have been shown to regulate anthocyanin synthesis in pitaya pulp (Zhou et al., 2020). StWRKY13 interacts with the gene promoters of *StCHS*, *StF3H*, *StDFR*, and *StANS*, significantly enhancing their activities and promoting anthocyanin biosynthesis in potato tubers (Zhang et al., 2021). MYBC1 and WRKY44 have been demonstrated to induce anthocyanin accumulation by binding to the promoters of the kiwifruit *F3’H* and *F3’5’H* genes, thereby strongly activating these promoters during kiwifruit coloring (Peng et al., 2020). In apple flesh, MdWRKY10 has been demonstrated to transcriptionally regulate *MdMYB10*, *MdF3’5’H*, and *MdUFGT*, thereby enhancing anthocyanin synthesis (Wang et al., 2024). FvWRKY50 has been demonstrated to directly upregulate the expression of *FvCHI* and *FvDFR* by binding to their respective promoters. However, FvWRKY50 can be phosphorylated by FvMAPK3 and subsequently degraded by ubiquitination at low temperatures, which delays anthocyanin accumulation in strawberries (Chen et al., 2023). Research found that *WRKY13* was significantly higher in white skin strawberries compared to red skin fruit (Xiao et al., 2024). In our study, ten RiWRKY TFs were downregulated during storage. *RiWRKY1* (*jg13940*) and *RiWRKY5* (*jg9885*) were significantly correlated with cyanidin-3-O-sophoroside (r > 0.9 and *p* < 0.01). In future studies, gene knockout or overexpression approaches will be employe to functionally validate the roles of key regulatory genes in anthocyanin synthesis.

In peach fruit, the presence of bHLH facilitates the interaction between PpNAC1 and BL, forming a heterodimer. This dimer enhances the transcriptional activation capacity of PpNAC1 on the *MYB10.1* promoter, thereby increasing the binding ability of MYB10.1 with the downstream key genes *PpDFR* and *PpUFGT* and ultimately promoting anthocyanin biosynthesis (Zhou et al., 2015). In red-fleshed apples, MdNAC14-like exhibited a significant negative correlation with anthocyanin content and was observed to bind to the promoters of *MdMYB9*, *MdMYB10*, and *MdUFGT*, thereby inhibiting their transcriptional activity and subsequently suppressing anthocyanin synthesis (Xu et al., 2023). In this study, the expression levels of *RiNAC2* (*jg28119*) and *RiNAC3* (*jg22665*) exhibited a trend similar to that observed in the changes in anthocyanin content during coloration (**Figure 5**). WGCNA revealed a significant and positive correlation between *RiNAC2*, *RiNAC3*, and cyanidin-3-O-sophoroside. Thus, the accumulation of anthocyanins may be related to RiNACs in raspberries. In conclusion, MYB, WRKY, bHLH, and NAC TFs may regulate the expression of structural genes involved in anthocyanin metabolism, thereby influencing the color variation observed in raspberries. Nevertheless, the precise molecular regulatory mechanisms of these TFs remain to be confirmed through further experimental investigation.

### 4.4 Plant hormones affect anthocyanin accumulation by controlling the expression of biosynthetic genes

The biosynthesis of anthocyanins is not only regulated by TFs and structural genes but also by plant hormones, including JAs, cytokinins (CK), ABA, and gibberellins (GA) (Li et al., 2022; Li and Ahammed, 2023). Exogenous application of JAs, SA, and ABA has been observed to induce anthocyanin accumulation in fruits, whereas exogenous application of auxins has been observed to have the opposite effect (Li et al., 2024; Gao et al., 2021). SA is a stress hormone synthesized via the PAL pathway (Xu et al., 2022). MdNPR1, a master regulator of SA signaling, interacts with and promotes the transactivation activity of MdTGA2.2, which binds to promoters and activates the transcription of genes involved in anthocyanin biosynthesis (*MdPAL*, *MdCHS*, and *MdDFR*) and regulation (*MdWRKY40*), thereby positively regulating anthocyanin biosynthesis (Li et al., 2024). In tea leaves, the exogenous application of MeSA has been demonstrated to increase flavonoid concentration by upregulating the expression of genes involved in flavonoid biosynthesis, including *CsPAL*, *CsC4H*, *Cs4CL*, *CsCHS*, *CsCHI*, *CsF3H*, *CsDFR*, *CsANS*, and *CsUFGT* (Li et al., 2019b).

IAA has been shown to facilitate fruit ripening and regulate the metabolism of other hormones (Liu et al., 2023). Overexpression of *MdIAA26* in apple calli and Arabidopsis thaliana promoted anthocyanin accumulation (Wang et al., 2020b). As illustrated in **Figure 6**, the expression levels of the eight RiSAs exhibited a corresponding increase in coloration, which aligns with the observed variation in anthocyanin content. Specifically, *RiSA3* (*jg24677*) exhibited a positive correlation with five DAMs (**Figure 8**; **Supplementary Table S10**). Most of the *RiIAAs* exhibited a downward trend during this process. Conversely, *RiIAA1* (*jg10266*) was negatively correlated with the five DAMs. The accumulation of ABA during the maturation of L. barbarum has been positively correlated with anthocyanin content (Li et al., 2019c).

MdbZIP44, an ABA-induced bZIP transcription factor in apple, has been demonstrated to promote anthocyanin accumulation by enhancing the binding of MdMYB1 to the downstream target genes promoters of *MdDFR* and *MdUF3GT* (An et al., 2018). In this study, the expression level of *RiABA2* (*jg38827*) was highest during the RBT-5 period, which was consistent with the cyanidin-3-O-sophoroside content. In contrast, the expression levels of *RiABAs* (*jg15624*, *jg15811*, and *jg602*) showed an inverse trend. It has been demonstrated that the F-box protein COI1 is essential for the JA-specific induction of the expression of the late anthocyanin biosynthetic genes *DFR*, *LDOX*, and *UFGT* (Shan et al., 2009). One study demonstrated that overexpression of *MdGA2ox7* effectively enhanced gene expression in anthocyanin synthesis and promoted anthocyanin accumulation (Yan et al., 2024). In this study, the expression levels of *RiGA1* (*jg12526*) and *RiGA2* (*jg21698*) declined during raspberry fruit coloration. Moreover, a negative correlation was observed between *RiGA1*, *RiGA2*, and DMAs, particularly cyanidin-3-O-sophoroside (**Figure 8**; **Supplementary Table S10**). These findings suggest that these hormones may play a role in anthocyanin synthesis during raspberry fruit color change. Nevertheless, further investigation is required to elucidate the regulatory mechanisms of the hormones associated with anthocyanin accumulation.

These findings offer significant potential for improving fruit storage, quality, and the bioengineering of high-anthocyanin crops. By employing CRISPR/Cas9-mediated gene editing or overexpression techniques, key TFs (such as RiMYB1) or structural genes (such as *RiANS1*) participating in anthocyanin biosynthesis can be precisely modulated. This approach enhances TF stability, maintains elevated anthocyanin levels, improves pigment stability, minimizes postharvest color deterioration, and boosts antioxidant capacity. Additionally, targeted exogenous treatments, such as ABA or SA application, could be implemented based on identified hormone-related genes to further promote anthocyanin accumulation. However, anthocyanin synthesis is highly sensitive to environmental factors (such as light, temperature). To address this limitation, key light- or temperature-responsive genes in raspberries could be engineered to stabilize production. High-anthocyanin crops obtained by above method would be able commercialized in terms of biotechnological applications or within nutritional industry. The ‘*Harrietez*’ cultivar is a widely grown commercial raspberry variety in temperate regions (North America, Europe, Asia). Due to its broad adaptability and genetic representativeness, the experimental results obtained in this study may extend to other raspberry cultivars of diverse geographical origins. Notably, compared to model plants (such as *Arabidopsis thaliana*, tobacco) or staple crops (such as rice, corn), raspberry lacks a mature, stable genetic transformation system. Thus, developing a robust and efficient transformation protocol for raspberry remains a critical research priority.

## 5 Conclusions

In this paper, transcriptome and metabolome analysis methods were used to analyze the biosynthesis of anthocyanins in postharvest raspberry fruits. The determination of total anthocyanin content showed that cyanidin was the main pigment accumulated in the reddening process of raspberry fruit. In addition, anthocyanin targeted metabolomics analysis showed that the main form of anthocyanin was cyanidin-3-O-sophoroside. WGCNA and correlation analysis of transcriptome and metabolome found that 9 structural genes, 19 TF and 14 hormone signaling genes were key candidate genes involved in the biosynthesis of cyanidin-3-O-sophoroside and play an important role in anthocyanin biosynthesis, identified as essential contributors to coloration in raspberry. These findings provide potential targets for molecular breeding to improve postharvest color stability in raspberries. For instance, manipulating *RiMYB3* or *RiNAC2* expression could delay anthocyanin degradation, enhancing visual quality during storage.

## Data Availability

The data presented in the study are deposited in the http://www.ncbi.nlm.nih.gov/bioproject/1255761 repository, accession number PRJNA1255761.
