# Supplementary figures and images for: Integrated metabolomics and transcriptomics unravel the biosynthZaesis mechanism of anthocyanin in postharvest red raspberry (Rubus idaeus L.)
Source: Front Plant Sci. 2025 May 13;16:1549458. doi: 10.3389/fpls.2025.1549458 (PMC12106431; doi:10.3389/fpls.2025.1549458)

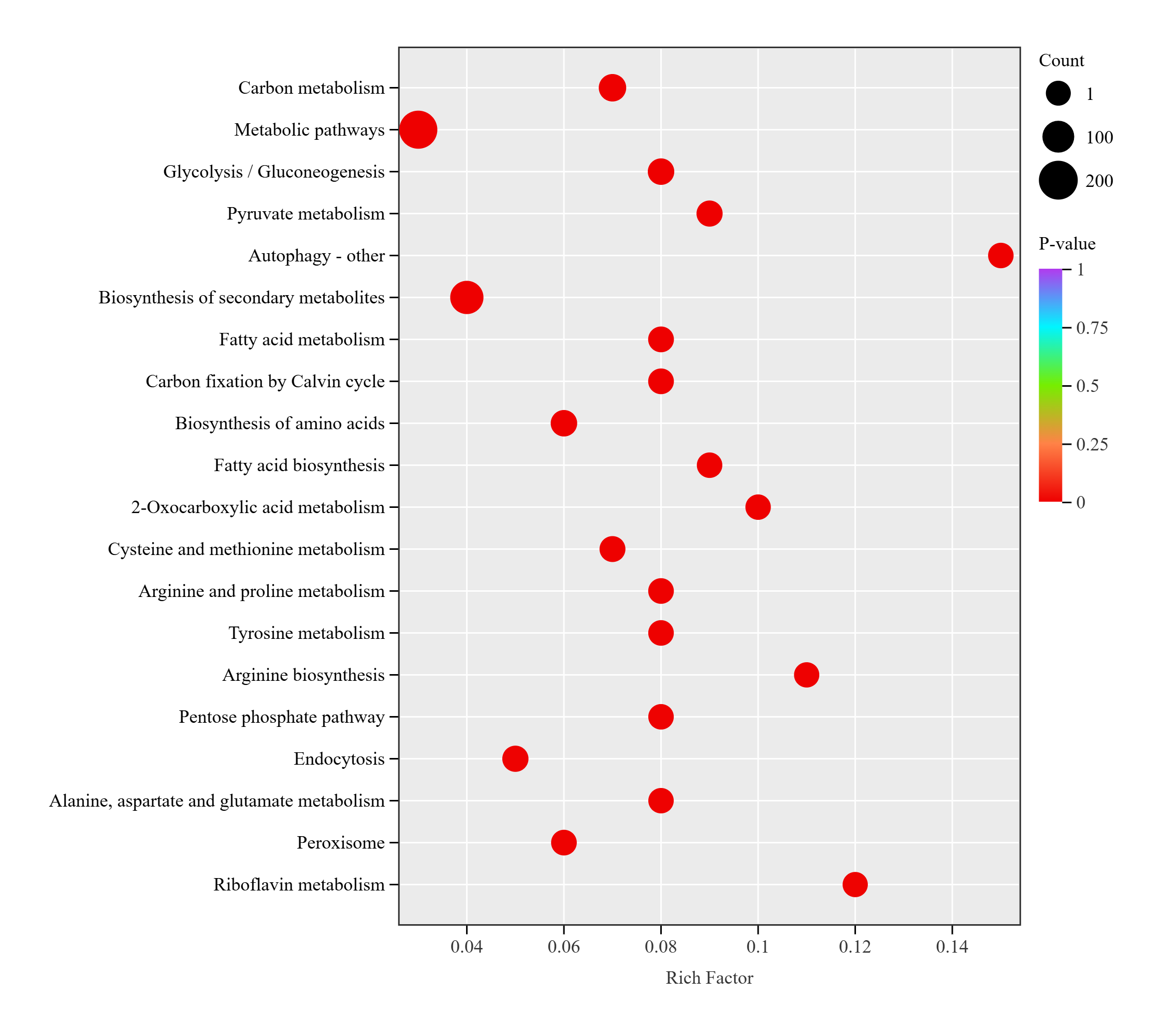

Supplement: Supplementary file 1 [file DataSheet1.zip › Supplementary Materials/Figure S1 KEGG enrichment of DEGs in turquoise module.png]

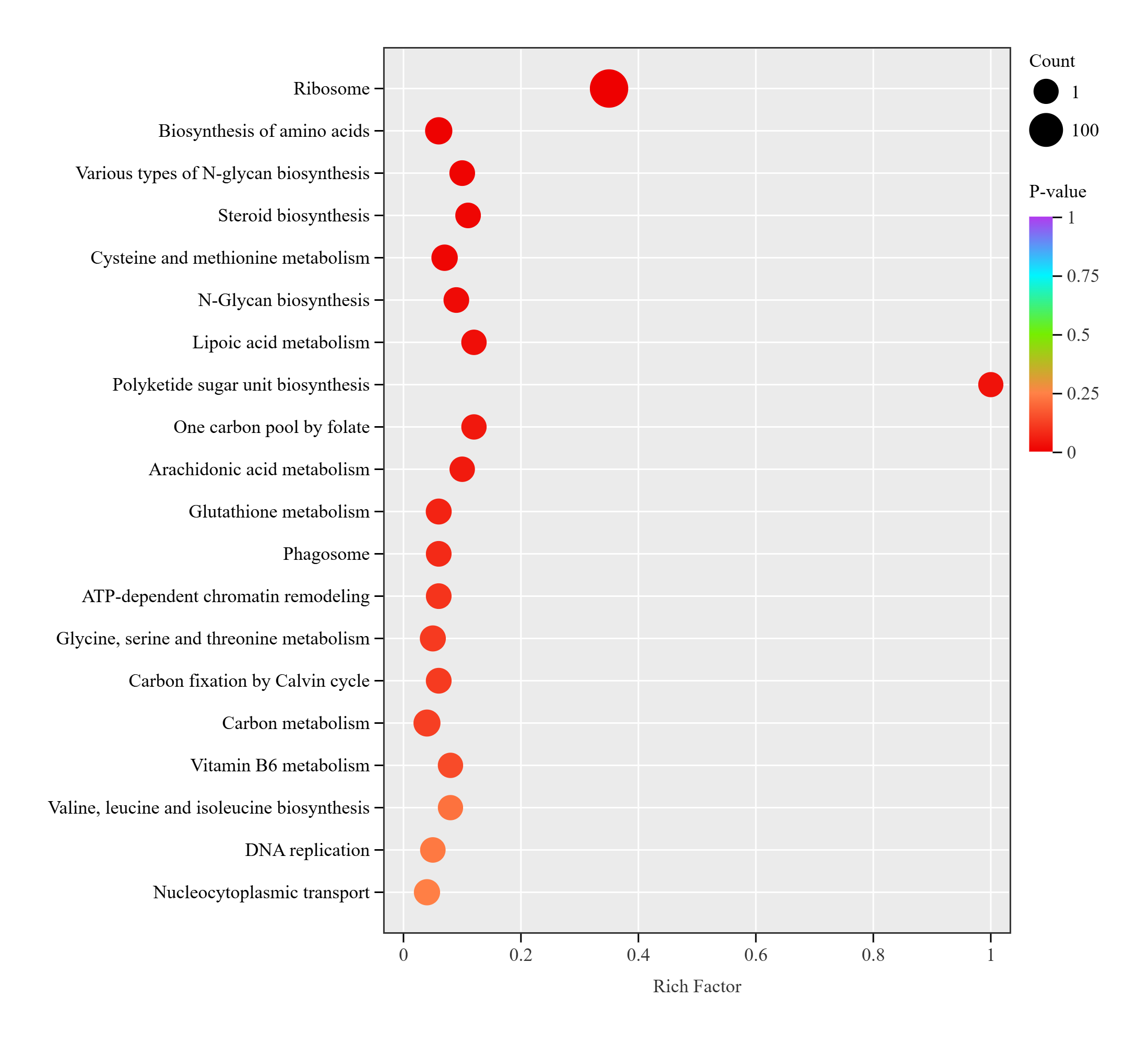

Supplement: Supplementary file 1 [file DataSheet1.zip › Supplementary Materials/Figure S2 KEGG enrichment of DEGs in blue module.png]

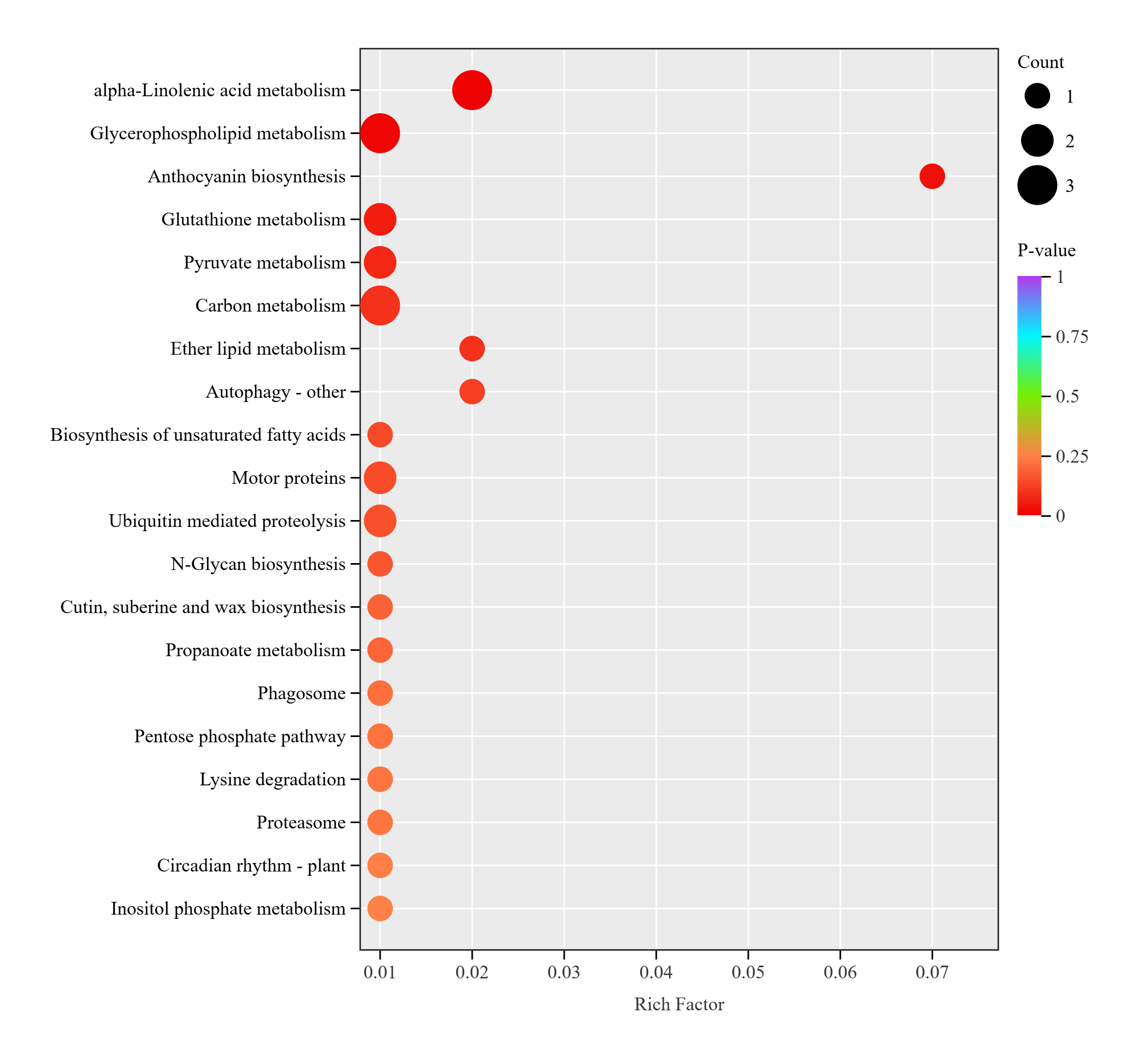

Supplement: Supplementary file 1 [file DataSheet1.zip › Supplementary Materials/Figure S3 KEGG enrichment of DEGs in pink module.png]

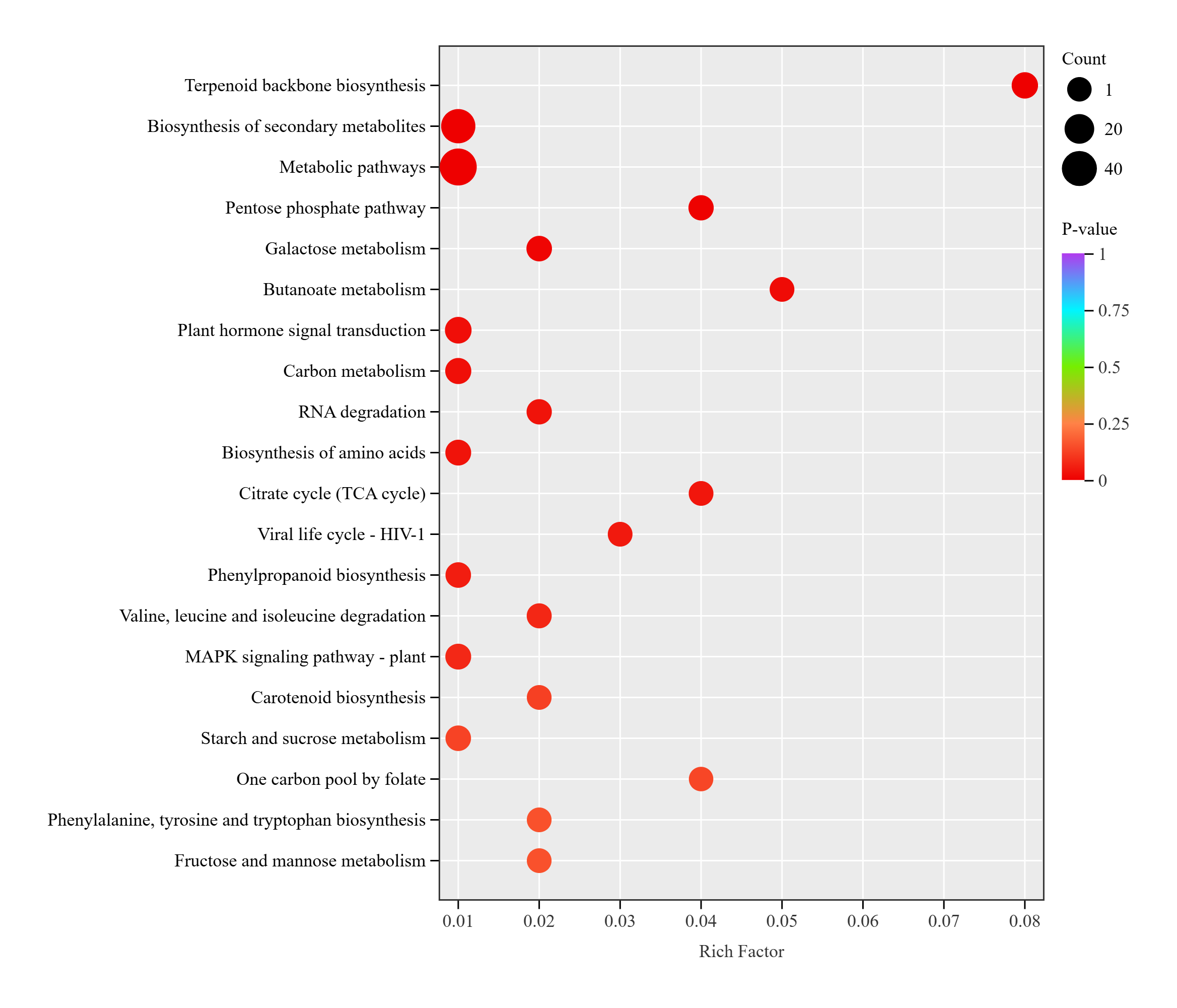

Supplement: Supplementary file 1 [file DataSheet1.zip › Supplementary Materials/Figure S4 KEGG enrichment of DEGs in yellow module.png]

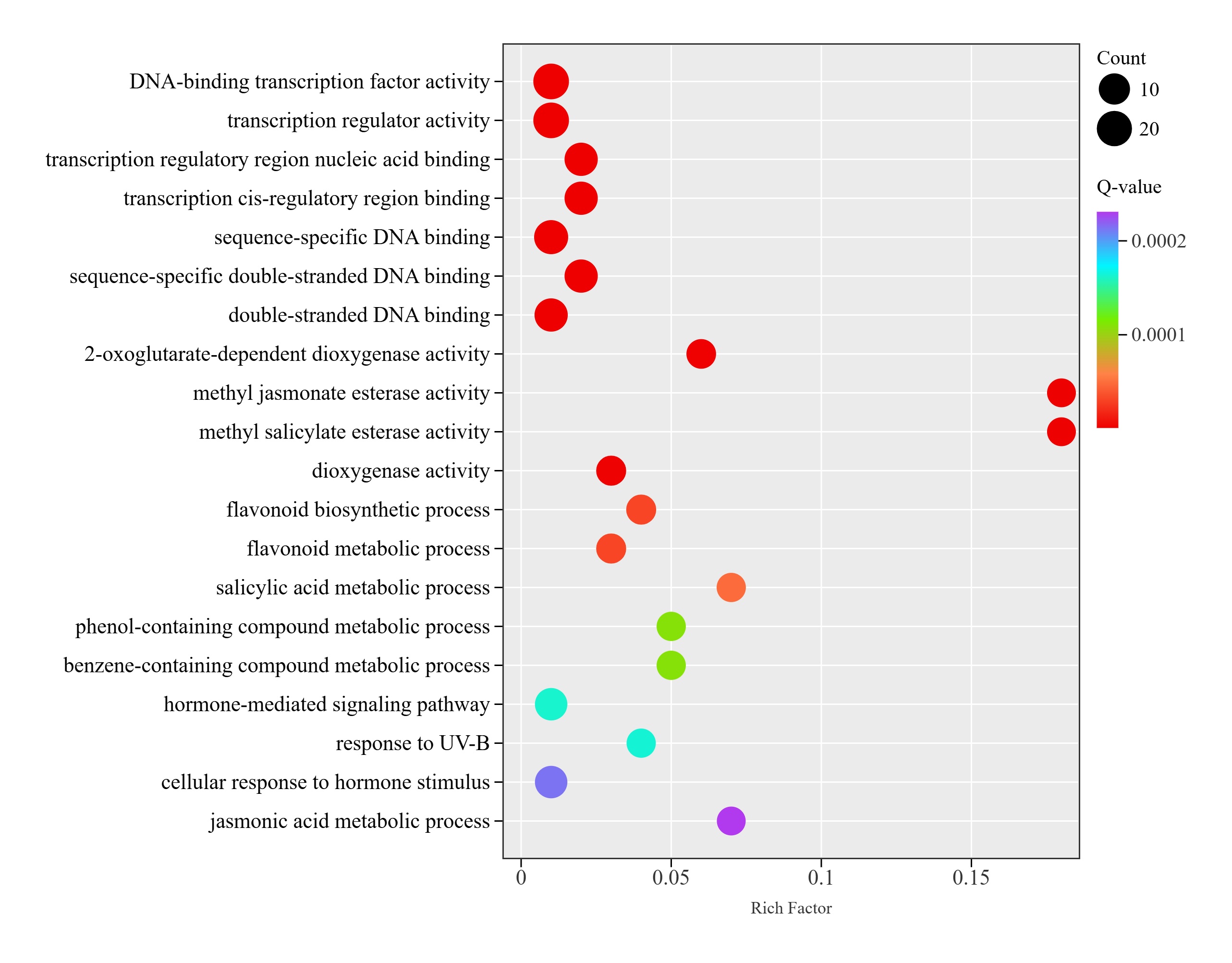

Supplement: Supplementary file 1 [file DataSheet1.zip › Supplementary Materials/Figure S5.png]
